# The aggregate proteome of *Caenorhabditis elegans* mitochondria implicates shared mechanisms of aging and Alzheimer’s disease

**DOI:** 10.3389/fnagi.2025.1713391

**Published:** 2026-01-13

**Authors:** Sonu Pahal, Akshatha Ganne, Meenakshisundaram Balasubramaniam, Sue T. Griffin, Robert J. Shmookler Reis, Srinivas Ayyadevara

**Affiliations:** 1 Bioinformatics Program, University of Arkansas at Little Rock and University of Arkansas for Medical Sciences, Little Rock, AR, United States; 2 Department of Geriatrics and Institute on Aging, University of Arkansas for Medical Sciences, Little Rock, AR, United States; 3 Central Arkansas Veterans Healthcare Service, Little Rock, AR, United States

**Keywords:** aging, Alzheimer disease, *Caenorhabditis elegans*, mitochondria, neurodegeneration, protein aggregation, proteomics, biomarkers

## Abstract

**Background:**

Mitochondrial dysfunction and protein aggregation are central features of brain aging and Alzheimer’s disease (AD). To define how AD seed proteins modulate these processes, we applied quantitative proteomics to sarkosyl-insoluble aggregates from *C. elegans* models of normal aging and from worms expressing human Aβ or Tau transgenes.

**Results:**

Normal aging produced a late-onset accrual of mitochondrial proteins within aggregates, implicating impaired energy metabolism and proteostasis collapse. Aβ expression caused a striking expansion and included glycolytic enzymes, tricarboxylic acid cycle components, ribosomal proteins, and trafficking factors, consistent with broad proteostatic and bioenergetic stress, largely overlapping with aging-associated species, yet advanced in onset. Tau expression yielded a smaller set enriched for cytoskeletal, vesicular, and nuclear pore components. Post-translational modifications (4-HNE adducts, phosphorylation, acetylation, methionine oxidation) revealed distinct trajectories: Aβ imposed early oxidative and phosphorylation burden, whereas Tau and aging showed midlife PTM peaks consistent with delayed proteostasis collapse. Cross-species comparison revealed 68 insoluble proteins shared between worm models and human AD brain aggregates. From these, 17 conserved metabolic, chaperone, and trafficking proteins were prioritized by network metrics and validated functionally: RNAi knockdowns aggravated paralysis or impaired chemotaxis, confirming their functional importance.

**Conclusion:**

These findings place mitochondrial proteome collapse at the center of aging and AD-seeded pathology, distinguish Aβ- and Tau-driven proteotoxic routes, and nominate a conserved panel of aggregation-prone proteins as mechanistic drivers and candidate biomarkers for early detection and intervention in AD.

## 1 Introduction

Mitochondrial impairment is a hallmark of both normal aging of the brain (Sutherland et al., 2021) and age-associated cognitive decline in AD (Esselun et al., 2021; Swerdlow, 2017). With age, brain mitochondria undergo structural and functional changes, including impaired biogenesis, accumulation of mtDNA deletions, oxidative mutations, membrane depolarization, respiratory chain malfunctions, disturbed Ca^2+^ balance, and a lowered threshold for permeability transition pore (PTP) opening (Gudenschwager et al., 2021; Jin and Cai, 2022; Cai and Tammineni, 2016). Complex I activity declines across the brain, most prominently in the cortex (Olesen et al., 2020; Weinert and Timiras, 2003). Defective Complex I in the electron transport chain (ETC) limits ATP generation and elevates ROS levels, which activate apoptotic and inflammatory pathways and increase pro-inflammatory cytokines (Sukhorukov et al., 2021; Bratić and Larsson, 2013). Given the high energy demand of the brain, reduced ATP and elevated ROS render neurons acutely vulnerable (Olesen et al., 2020; Weinert and Timiras, 2003). Age also reduces mitochondrial biogenesis and mitophagy (clearance of damaged mitochondria) (Chistiakov et al., 2014; Varghese et al., 2020), leading to accumulation of damaged mitochondria, an early feature of AD progression (Chistiakov et al., 2014; Varghese et al., 2020). With age, mitochondria of hippocampal neurons are especially susceptible (Cai and Tammineni, 2016; Olesen et al., 2020; Cilleros-Holgado et al., 2023) and mitochondrial damage is also seen in glial cells, particularly microglia, with high levels of mtDNA lesions (Cilleros-Holgado et al., 2023). These combined alterations establish a neurotoxic milieu. Mitochondrial dysfunction precedes clinical symptoms of AD (Pahal et al., 2025; D’alessandro et al., 2025), suggesting a causal role in Alzheimer’s pathogenesis. ATP depletion, ROS elevation, impaired ionic balance, reduced biogenesis, and neuroinflammation may jointly support a positive-feedback loop to drive neuronal deterioration.

Protein aggregation, long recognized as a hallmark of aging (Ayyadevara et al., 2016b; Cuanalo-Contreras et al., 2023), is a diagnostic of neurodegenerative diseases (NDs)—e.g. Aβ and Tau deposits in AD (Ross et al., 2015; Ayyadevara et al., 2016a), ɑ-synuclein in PD (Spillantini et al., 1998), and mutant HTT in Huntington’s disease (Aufschnaiter et al., 2016). Such aggregates disrupt proteostasis and trigger cell death pathways. Mitochondrial proteins are frequent components of these inclusions. Misfolded ETC subunits are reported in PD model (Castro et al., 2012), and *α* -synuclein interacts directly with Complex I of the mitochondrial ETC (Devi et al., 2008). ATP synthase in Complex V colocalizes with *α*-synuclein, promoting PTP opening and mitochondrial swelling (Bonora et al., 2020). Aβ and α-synuclein also interact with PTP-associated proteins, including VDAC, ANT, and cyclophilin D (Bonora et al., 2020). Chaperones are likewise represented: HSP60 (mitochondria-enriched, not exclusive) and HSP90 family members (not mitochondrial-specific) have been detected in PD Lewy bodies (Ingram and Chakrabarti, 2016; Kalia et al., 2010), and α-synuclein traps HSP10 precursors, reducing HSP60/HSP10 function in aging neurons (Krämer et al., 2020). In Drosophila and other models, loss of the mitochondrial protease HtrA2 leads to accumulation of misfolded proteins, underlining the role of mitochondrial quality control (Castro et al., 2012). Core metabolic enzymes are also vulnerable: the α-ketoglutarate dehydrogenase complex (KGDHC) is enriched in AD mitochondrial aggregates (Bender et al., 2011), and SOD1 has been reported among mitochondrial inclusions in disease models(Liu et al., 2004; Pasinelli et al., 2004) Our neural network analysis estimated that ~20% of the most AD-enriched aggregate proteins are mitochondrial (Balasubramaniam et al., 2023). Together, these reports establish mitochondrial aggregation as a recurrent and mechanistically implicated feature of aging and neurodegeneration.

Proteostasis governs protein folding, trafficking, and clearance, and underpins mitochondrial biogenesis, interorganellar exchange, and mitophagy (Cuanalo-Contreras and Moreno-Gonzalez, 2019; Moehle et al., 2018). Even misfolding of a single ETC subunit can block assembly or proper localization to the inner membrane, while mitochondrial ROS can further modify proteins by oxidation, nitration, carbonylation, or cleavage. Collapse of proteostasis within mitochondria leads to aggregate accumulation, which in turn impairs proteostasis efficiency and accelerates neurodegenerative decline (Köhler et al., 2015).

Under proteotoxic stress, the mitochondrial unfolded protein response (UPR^mt^) is activated: HSP-6 (HSP70 family) facilitates ATFS-1 translocation to the nucleus, where ATFS-1 cooperates with UBL-5 and DVE-1 to induce chaperones and proteases (Hoppe and Cohen, 2020). UPR^mt^ and proteases maintain folding homeostasis in the inner membrane, matrix, and intermembrane space, while the ubiquitin–proteasome system functions at the outer membrane. Disaggregases such as HSP104 transfer misfolded proteins from other compartments to mitochondria, where proteases including LON degrade them (Bar-Ziv et al., 2020a; Bar-Ziv et al., 2020b; Rai et al., 2022).

Protein aggregation and mitochondrial dysfunction act in a positive feedback loop: stress and protein damage or modifications accelerate aggregation, while aggregates further impair mitochondrial biogenesis and function. This vicious cycle drives neurodegeneration. In the present study, we sought mitochondrial proteins most impacted by normal aging or by expression of AD seed proteins (human Aβ and Tau) in *C. elegans*. By identifying mitochondrial proteins not previously known to aggregate, and the pathways perturbed by their sequestration, we aimed to reveal early mechanistic drivers of pathology and novel biomarker candidates for AD.

## 2 Materials and methods

### 2.1 *Caenorhabditis elegans* strains

Three *C. elegans* strains employed in this study were provided by the Caenorhabditis Genetics Center (CGC; Minneapolis, MN, USA): SJ4103 [zcIs14 [myo-3: GFP(mit)]] as a normal aging model that expresses Green Fluorescent Protein in muscle mitochondria; CL2355 [dvIs50 [pCL45 (snb-1):Abeta 1–42:3’ UTR(long) + mtl-2:GFP]] with pan-neuronal expression of human Aß_1–42_ peptide; VH255 [hdEx82 [F25B3.3:Tau352(WT) + pha-1(+)]] with pan-neuronal expression of native human Tau.

### 2.2 Age synchronization of *Caenorhabditis elegans* cohorts

All strains were grown on NGM agar Petri plates overlaid with lawns of *E. coli* (strain OP50) and maintained at 20 °C. Healthy adult worms were collected and washed with S-buffer to remove bacteria and larvae. A lysis solution was prepared comprising 1.0% (w/v) sodium hypochlorite (diluted from commercial bleach, 5.25–6.0% NaOCl; Clorox Regular) in 1.0 N NaOH. Centrifuge tubes containing worms and lysing solution were gently agitated at 20 °C until the worm cuticle dissolved (~5 min). Immediately, the bleach solution was quenched with S-buffer and eggs were washed ≥3x in S-buffer to remove residual lysing solution. Eggs were collected by centrifugation and placed on OP50 plates at 20 °C, resulting in age-synchronized cohorts. Day-1 adult CL2355 worms (~3.5 days post-hatch) were upshifted to 25 °C to induce Aβ_1–42_ expression. We added 5-fluoro-2′-deoxyuridine (FUDR) at a very low final concentration (2 μM) to slow the growth of any larval progeny. Worms were transferred to fresh OP50 plates every day. Age-synchronized worms were collected, washed thoroughly with S-buffer to remove bacteria, and snap-frozen at adult days 1 (day 3 post-hatch), 5 (day 7 post-hatch), and 9 (day 11 post-hatch).

### 2.3 Mitochondrial pulldown and aggregate preparation

Snap-frozen worms at three ages were ground to a powder in a dry ice–cooled mortar and pestle. Pellets from each sample were suspended in 1 × pre-extraction buffer, prepared as a 1:10 dilution of the stock buffer supplied with the Abcam Nuclear Extraction Kit (ab113474) and supplemented with phosphatase and protease inhibitors for mild disruption of the plasma membrane. Suspensions were incubated at 4 °C for 10 min, then disrupted and resuspended using a Dounce homogenizer, and spun at 600 × g for 1 min to remove large debris.

Total protein was quantified for each sample using Bradford reagent (Bio-Rad; Hercules, CA, USA), and equal amounts of protein were incubated overnight at 4 °C for gentle agitation with Dynabeads™ (MA5-12242, ThermoFisher) coated in antibody to TOMM22 (Abcam, ab57523). This “immuno-pulldown” (IP) targeting an outer mitochondrial membrane protein, TOMM22, replaced centrifugation to preserve mitochondrial integrity.

Proteins from each fraction were suspended in 0.1 M HEPES buffer (pH 7.4) containing 1% v/v sarkosyl and 5 mM EDTA, followed by centrifugation at 4 °C for 30 min at 100,000 × g. Sarkosyl-insoluble fractions were suspended in Laemmli buffer [66-mM Tris-HCL, pH 6.8, containing 50-mM dithiothreitol and 2% v/v sodium dodecyl sulfate (SDS)] and boiled for 5 min. Insoluble aggregate aliquots of each sample were resolved by electrophoresis on 4–20% polyacrylamide gradient gels, which were stained with Coomassie blue for visualization and protein quantitation. Robotically excised 1-mm slices were digested with trypsin prior to analysis by LC–MS/MS.

### 2.4 Mitochondrial purity assessment by western blotting

Proteins in the TOMM22-bound mitochondrial fraction and the unbound cytosolic fractions from *C. elegans* strains SJ4103 and CL2355 were resolved directly by SDS–PAGE. Protein was transferred to PVDF membranes; transfer was verified by staining with Ponceau S (Thermo Scientific™), which was removed in 0.1% NaOH before immunodetection. Membranes were probed with mitochondria-specific antibodies to VDAC-1 (Abclonal A19707), PDHB (Invitrogen PA5-27989), OGDH (Cell Signaling Technology E1W84), contrasted with eEF-2 (Abclonal, A9721) which is mostly cytosolic. Blots were washed 4x in TBS containing 0.1% Tween-20 (TBST), and then incubated with HRP-conjugated anti-rabbit secondary antibody (Cell Signaling, 7,074) for 1 h. After washing the blot with TBST, immunoreactive signal was visualized on a Bio-Rad imager (cat.# 12,003,154) using ClarityMax ECL western blotting substrate (Biorad 1705060S).

### 2.5 Protein identification and validation with scaffold

MS/MS spectra were searched against the *C. elegans* UniProtKB/SwissProt reference proteome (release December 2022) using Mascot (Matrix Science, v2.8). Search parameters allowed a precursor tolerance of 10 ppm and fragment ion tolerance of 0.6 Da. Carbamidomethylation of cysteine was set as a fixed modification, with methionine oxidation included as an optional variable. Peptide and protein identifications were reviewed in Scaffold 5 (Proteome Software, Portland, OR). Protein identifications were accepted only if supported by ≥2 unique peptides while restricting the global protein FDR to <1%.

### 2.6 PEAKS™ analysis of post-translational modifications (PTMs)

For independent PTM assessment, raw spectra were also searched using PEAKS Studio X + (Bioinformatics Solutions Inc.) with an expanded set of dynamic modifications including lipid peroxidation adducts (4-hydroxynonenal (4-HNE) on Cys/His/Lys/Arg), lysine acetylation, serine/threonine/tyrosine phosphorylation, and methionine oxidation. High-confidence PTM assignments were accepted at <1% peptide-level FDR and PEAKS local confidence score ≥ 20.

### 2.7 Gene ontology (GO) enrichment analysis of proteins

Proteins were functionally annotated for molecular function (MF), cellular component (CC), biological process (BP), and KEGG pathways using g: Profiler’s g: GOSt tool (Kolberg et al., 2023). The analysis was restricted to curated *C. elegans* GO terms with the Benjamini–Hochberg false-discovery rate (FDR) limited to <0.05. Enriched terms were visualized with bubble plots, and pathway diagrams were generated in SRplot (Tang et al., 2023).

### 2.8 Cross-species proteomics integration and functional validation in *Caenorhabditis elegans* AD models

Proteins preferentially aggregated in *C. elegans* AD models were identified by comparison with age-matched insoluble proteomes. Human orthologs were assigned using g: Profiler’s g: Orth (Kolberg et al., 2023). Ortholog IDs were then cross-referenced with (a) our prior human AD insoluble-aggregate proteome (Ayyadevara et al., 2016a), and (b) AD serum proteomics data from Dey et al. (2019). Candidate lists were ranked by fold-change in AD hippocampal vs. age-matched control aggregates, and by influence score and degree (number of direct partners by intra-aggregate crosslinking) in Aβ- and Tau-IP interactomes (Ayyadevara et al., 2016a). Top-ranked proteins were validated by RNAi knockdown in *C. elegans* strains CL2355 and VH255, as described below.

### 2.9 Chemotaxis assay

Transgenic *C. elegans* strain CL2355, expressing human Aβ_1–42_ in all neurons, was grown at 20 °C with ample *E. coli* (OP50) bacteria, and adult worms were lysed to release eggs and, thus, to initiate a synchronous-aging cohort. Eggs were then placed on 100-mm diameter NGM-agar Petri dishes seeded with bacteria expressing an RNAi segment or control bacteria carrying the empty vector (Feeding Vector, FV). Aβ_1–42_ expression was induced by upshifting to 25 °C, and 2-μM FuDR was added to plates to impede the growth of progeny at the larval stage 4 (L4). Worms were collected and washed free of bacteria (3–4 × in S buffer) at day 5 post-hatch. Assay plates were kept at 20 °C, and motility towards 1-butanol was scored after 2 h. Each experiment was performed ≥ 3 times. The ‘Chemotaxis Index’ (CI) was calculated for a normalized response.

### 2.10 Paralysis assay

Cohorts of *C. elegans* strain VH255, expressing human Tau in all neurons, were age-synchronized as described above and maintained at 20 °C. Worms were transferred to new plates, and 2-μM FuDR was added at the L4 stage to maintain a healthy and age-synchronized cohort. Motile and paralyzed (no movement in response to touch) worms were scored on adult day 10 (day 12 post-hatch). The experiment was performed three times.

### 2.11 Statistical analysis

Statistical analysis was performed using Graph Pad Prism (GraphPad Software, CA) and VassarStat online modules. Unless stated otherwise, values are reported as mean ± SEM. Between-group differences for behavioral assays (chemotaxis index, paralysis) were assessed by two-tailed *t* tests, with Benjamini–Hochberg adjustment across gene panels where indicated. Protein identifications were accepted in Scaffold 5 at protein-level FDR < 1% and ≥2 unique peptides per protein. PTM site calls from PEAKS Studio X + were filtered at peptide-level FDR < 1% with local confidence ≥ 20. Functional enrichment (GO, KEGG) used g: Profiler (g: GOSt) with FDR < 0.05 unless noted. Early aggregation was defined *a priori* as ≥ 2-fold enrichment versus age-matched normal aging at adult day 1 or day 5. Software versions match those listed in Methods. Significance of enrichment or depletion of individual proteins with age was derived from Fisher Exact tests on 2×2 tables relative to total aggregate-protein abundances at the same ages, without Bonferroni correction for multiple endpoints.

## 3 Results

**Figure 1:**
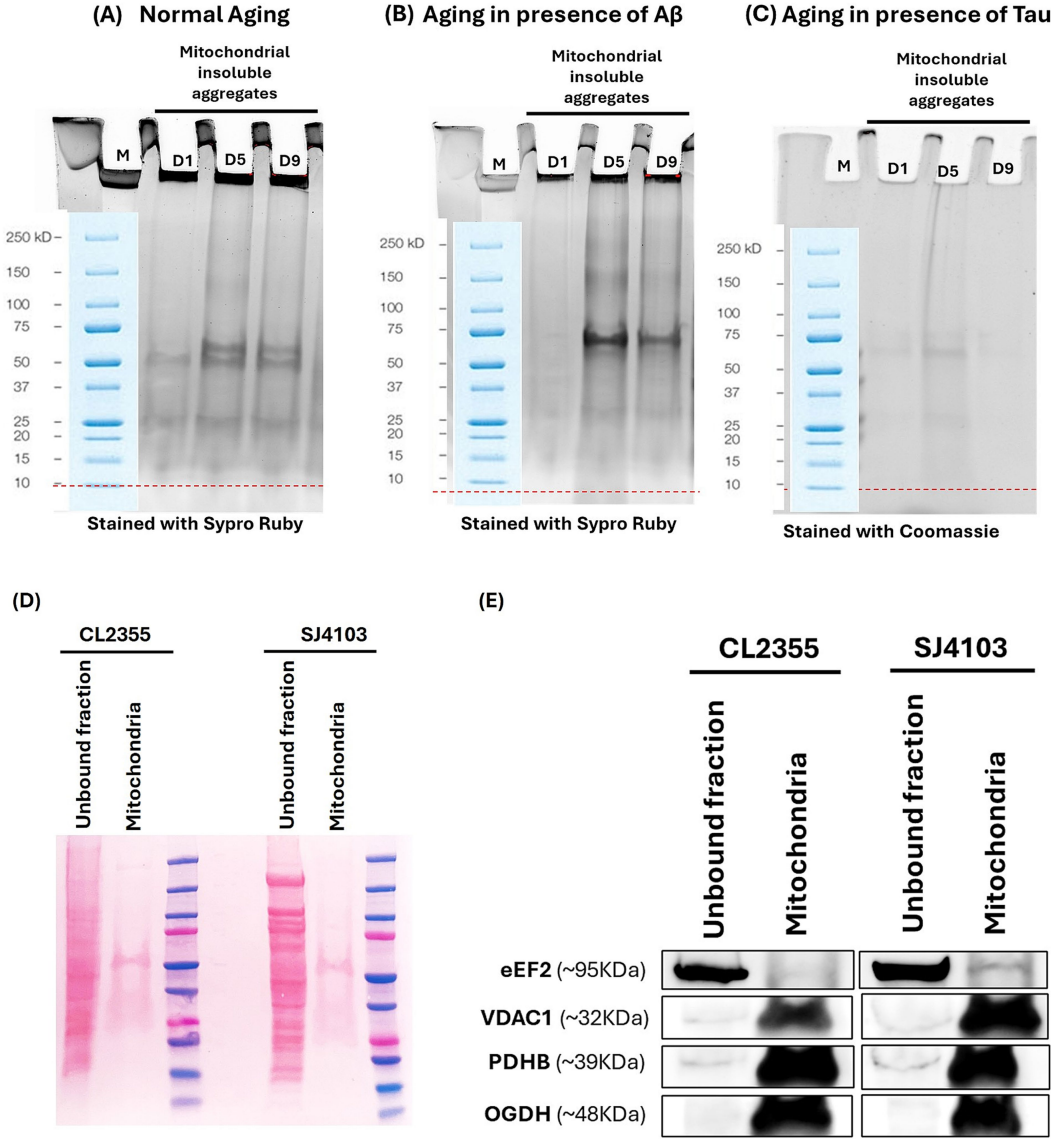
Insoluble mitochondrial aggregates and validation of mitochondrial purification. **(A-C)** Sarkosyl-insoluble mitochondrial aggregates isolated from three *C. elegans* strains: SJ4103 (normal aging), CL2355 (Aβ-expressing), and VH255 (Tau-expressing) were resolved by acrylamide-gel electrophoresis. For each strain, fractions from adult days 1, 5, and 9 were analyzed in parallel (M: molecular-weight marker). Gels were stained with Sypro Ruby (SJ4103, CL2355) or Coomassie (VH255) to visualize total insoluble protein. **(D)** Ponceau S stained PVDF membranes confirm protein transfer in the TOMM22-eluted mitochondrial fraction and the corresponding unbound fraction from SJ4103 and CL2355 worms, which document the presence of transferred protein in each lane prior to immunodetection. **(E)** Immunoblot assessment of mitochondrial enrichment. Mitochondrial fractions (TOMM22-eluted) contain VDAC-1, PDHB, and OGDH, consistent with mitochondrial outer-membrane and matrix localization, with little or no detectable eEF-2. The reciprocal pattern in the corresponding unbound fractions confirms effective separation of mitochondria.

We isolated sarcosyl-insoluble aggregates from mitochondria of three strains, and compared their protein profiles during normal aging (Figure 1A) and in the presence of Aβ (Figure 1B) or wt-Tau seed proteins (Figure 1C). The purity of mitochondria was supported by the presence of mitochondrion-specific proteins such as VDAC-1, PDHB, OGDH only in mitochondrial fractions, contrasted to their relative absence from post-mitochondrial cytoplasm by western blot probing (compare Figures 1D,E, showing Ponceau S staining of the membrane). The predominantly cytoplasmic protein eEf-2 was observed in cytoplasm but was absent from proteins isolated from the mitochondrial fraction (Figure 1E).

### 3.1 The number of mitochondrial insoluble-aggregate proteins changes with age and in the presence of Aβ or Tau transgenic proteins

**Figure 2:**
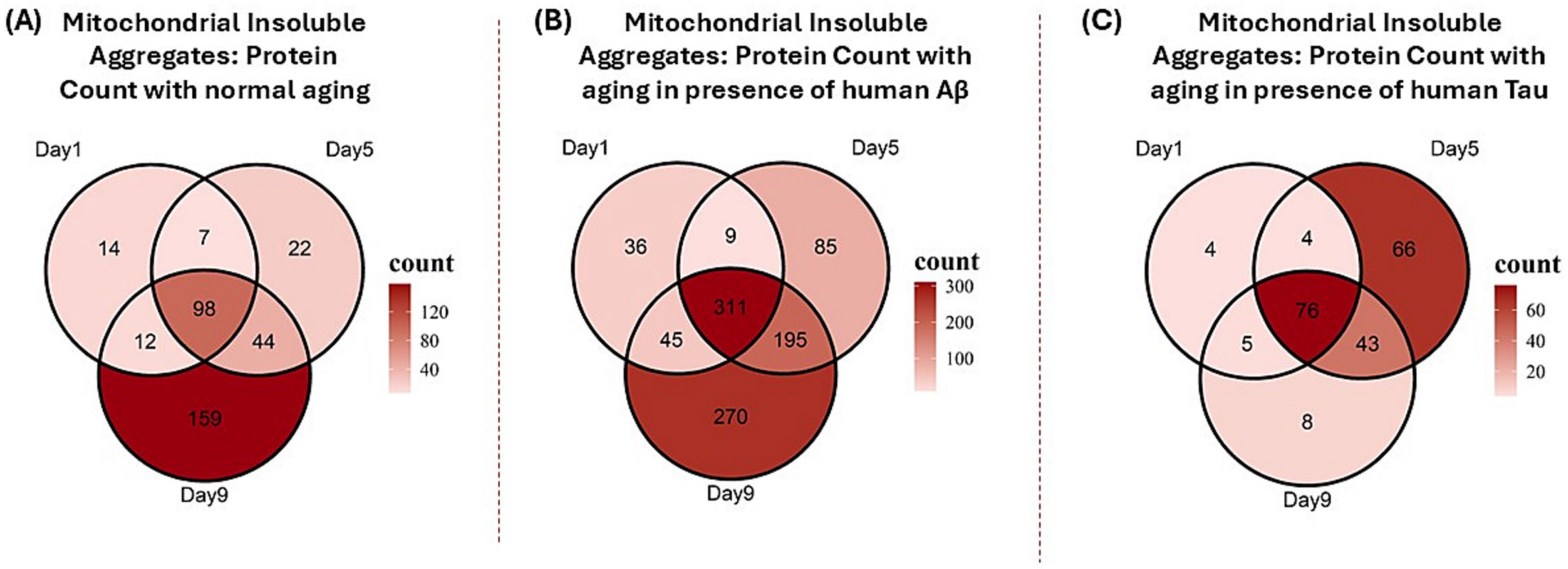
Venn diagrams showing distribution of proteins in mitochondrial insoluble aggregates **(A)** with normal aging, **(B)** aging in the presence of Aβ, and **(C)** aging in the presence of Tau.

During normal aging, mitochondrial insoluble aggregates increased progressively from adult day 1 to day 9. In nematodes expressing Aβ, insoluble aggregates were elevated at each adult age relative to normal aging and also rose with age from day 1 to day 9. In contrast, Tau expression produced significantly fewer insoluble-aggregate proteins at any age relative to normal or Aβ-associated aging, peaking at day 5 (Figure 2). This profile echoes prior work from the Morimoto group, who reported that polyglutamine aggregates in a *C. elegans* model of Huntington’s disease accrue early in adulthood, peaking near adult days 5–6 at 20 °C (Morley et al., 2002). We found that Tau-driven pathology shifts the aggregation trajectory to an earlier, lower-amplitude peak than either Aβ- or age-associated pattern.

**Figure 3:**
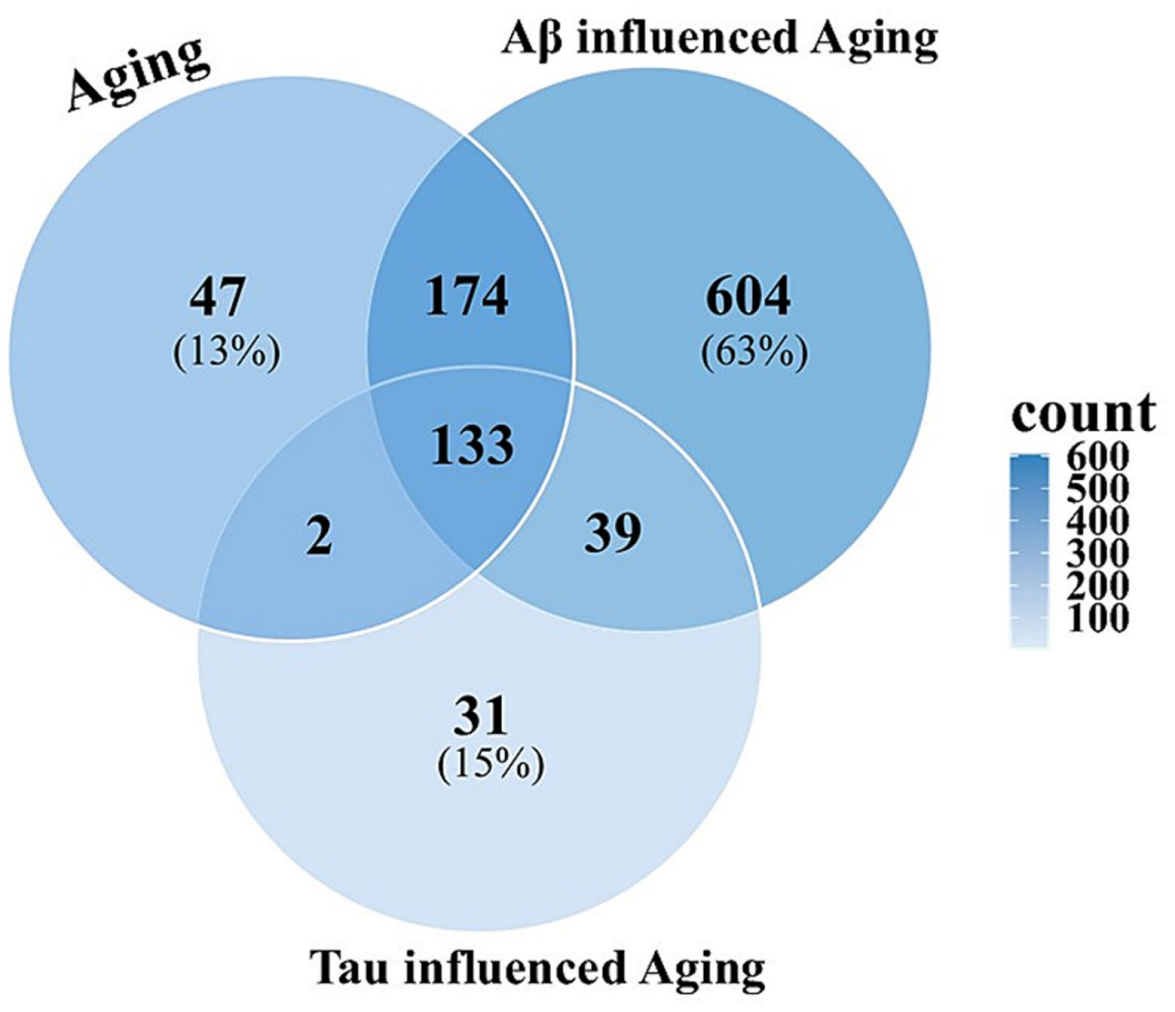
Venn diagram representing total protein count of unique and shared proteins in mitochondrial insoluble aggregates with Aging, Aging with Abeta, and Aging with Tau, at 3 *C. elegans* ages.

Nematodes aging normally accumulate 356 proteins in mitochondrial insoluble aggregates, relative to young adult worms. Aging worms that express human Aβ accrued 950 proteins, while those expressing human Tau accumulated 205 proteins (Figure 3). A total of 133 proteins were identified as common to all three conditions, evidently unaffected by the expression of AD seed proteins. We noted a substantial overlap (307 proteins) between aging with and without amyloid-beta (Aβ) expression, while a smaller overlap was observed between aging with and without Tau expression (Figure 3). GO enrichment of the full insoluble proteomes for aging, Aβ, and Tau is provided as Supplementary Figure 1.

### 3.2 Core aggregation-prone proteins common to aging and Alzheimer’s

**Figure 4:**
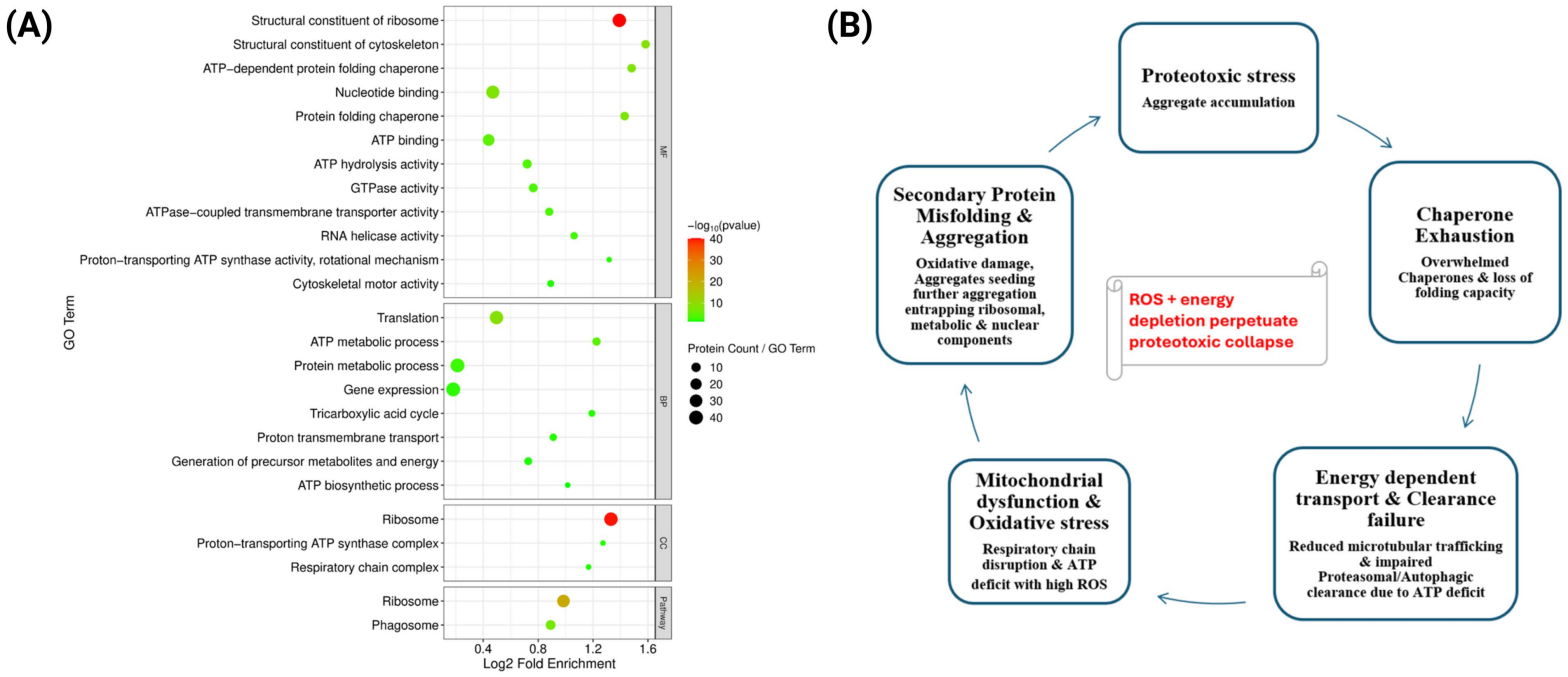
**(A)** Bubble plot representing GO enrichment analysis for age-associated core aggregation proteins with bubble size representing gene count and color showing adjusted *p*-value <0.05 (Benjamini-Hochberg correction). MF: Molecular Function, BP, Biological Processes; CC, Cellular Component. Panel **(B)** illustrates the vicious cycle of proteotoxic stress, chaperone exhaustion, failure of energy-dependent processes, mitochondrial dysfunction, oxidative stress, protein misfolding, and amplified proteotoxic stress, all of which feed back to increased aggregate burden.

The 133 proteins shared across all three conditions represent a core set most vulnerable to age- and AD-associated aggregation. Annotation-term (GO) enrichment highlights key sites, metabolic processes, and pathways that impact the functional integrity of neurons. The most enriched terms were associated with mitochondrial energy metabolism, including respiratory chain complex, ATP biosynthesis, proton-transporting ATP synthase complex, and the tricarboxylic acid cycle, underscoring the prominence of metabolic enzymes in the aggregate proteome. In parallel, terms for translation and proteostasis were highly over-represented: ribosome, ribosomal constituents, translation, ATP-dependent chaperone activity, protein folding, and nucleotide binding. Enrichment also extended to structural terms such as cytoskeletal organization, reflecting age- and AD-associated collapse of large macromolecular assemblies. Taken together, the shared proteins indicate a conserved signature of age- and disease-driven aggregation, dominated by pathways in bioenergetics, proteostasis, translation, and cytoskeletal integrity (Figure 4).

### 3.3 Dynamics of post-translational modifications to mitochondrial insoluble aggregates during normal and Alzheimer’s-like ageing of *Caenorhabditis elegans*

#### 3.3.1 4-HNE adducts (at CHLR residues), secondary to lipoperoxidation

**Figure 5:**
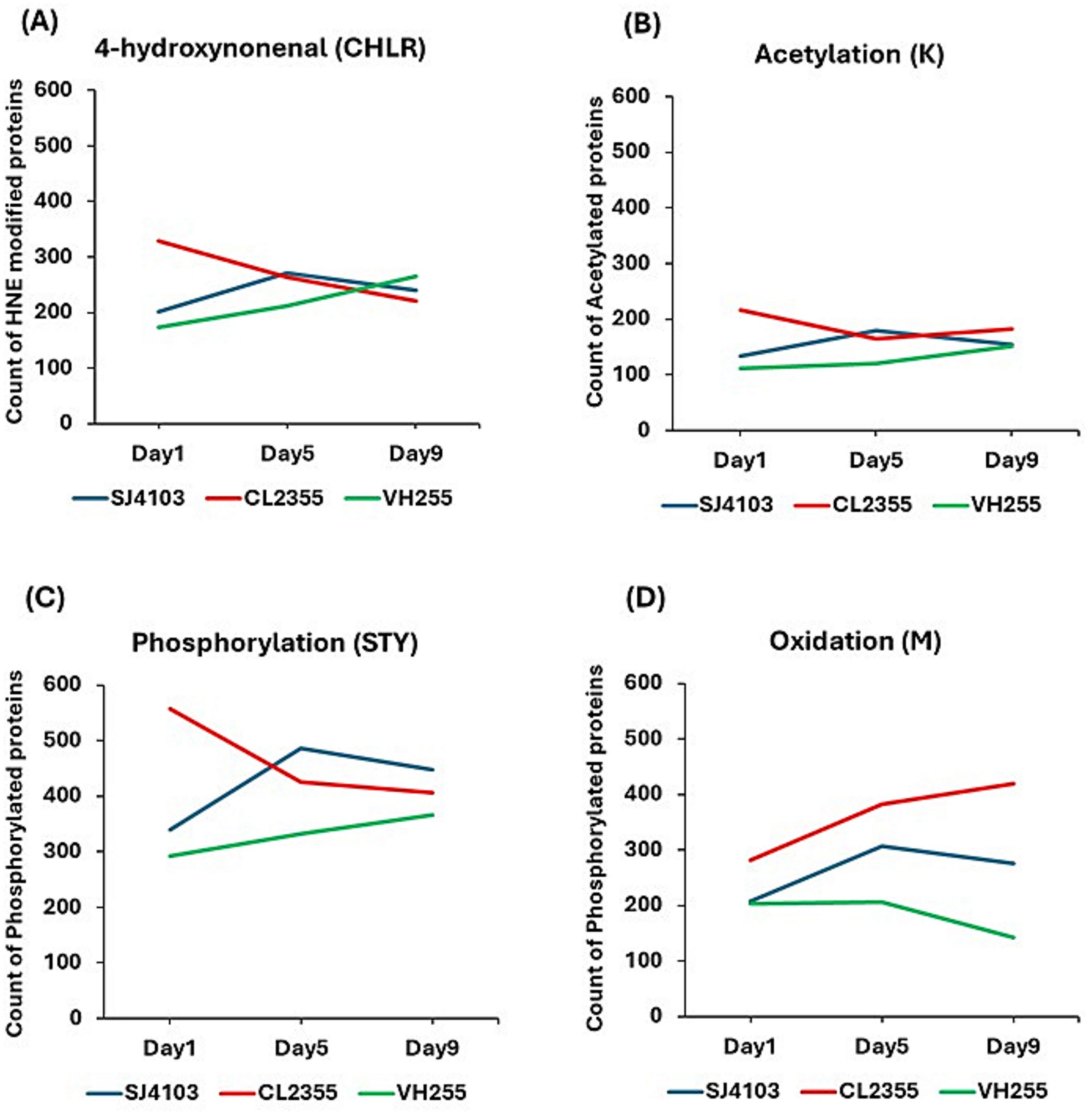
Post-translational modification dynamics in mitochondrial insoluble aggregates from *C. elegans* models of normal and AD-like aging. Line graphs show total counts at adult days 1, 5, and 9 for: **(A)** proteins adducted with 4-hydroxynonenal (CHLR), reflecting lipoperoxidation damage. **(B)** proteins acetylated on lysine (K); **(C)** proteins phosphorylated on serine, threonine, and/or tyrosine (STY); and **(D)** oxidation of methionine (M). Blue: SJ4103 (normal-aging control). Red: CL2355 (Aβ model). Green: VH255 (Tau model).

4-HNE is an abundant product of lipid peroxidation chain reactions. It is highly reactive, and gives rise to HNE-modified proteins observed in all strains (Figure 5A). At day 1, Aβ-expressing worms (CL2355) carried the greatest burden (329 sites), compared with normal-aging controls (201 sites in strain SJ4103) or Tau-expressing worms (173 sites in VH255). In normal aging, HNE adducts rose to a midlife peak (271 at day 5) before declining (240 at day 9). Tau animals followed a similar trajectory (212 → 265). In contrast, Aβ worms showed their maximum at day 1 (329), falling steadily through day 9 (221). By day 9, the three strains had similar adduction levels.

#### 3.3.2 Phosphorylation (STY)

Because we had previously observed phosphorylated proteins to be enriched 3.2- to 3.5-fold for AD hippocampal aggregates relative to normal controls (Ayyadevara et al., 2016a), we next analyzed phosphorylation sites (serine/threonine/tyrosine) in aggregated proteins. Phosphorylated residues were most numerous in Aβ aggregates (557 sites at day 1), exceeding normal aging (340) and Tau (293). Numbers fell with age in Aβ worms (425 → 406), whereas both normal (340 → 487 → 448) and Tau (293 → 333 → 367) strains peaked at midlife before partial decline (Figure 5B). Thus, Aβ drives an early hyper-phosphorylation phenotype.

#### 3.3.3 Acetylation (K)

Lysine acetylation was also assessed, to pursue our earlier finding of 3.3-fold more such sites in AD vs. control amyloid aggregates and 2.8-fold more in Tau aggregates (Ayyadevara et al., 2016a). Consistent with the human tally, acetylated peptides were likewise enriched in Aβ worms (217 at day 1) relative to normal aging (134) or Tau (111). Unlike phosphorylation, acetylation patterns fluctuated only modestly over time, with normal and Tau worms showing modest increases by day 9 (154 and 152, respectively), while Aβ worms remained elevated (182; Figure 5C).

#### 3.3.4 Methionine oxidation (M)

Proteins oxidized at methionine residues were previously reported as the most abundant oxidative modification in AD-specific hippocampal aggregates, nearly three-fold higher than in normal controls (Ayyadevara et al., 2016a). The aging profile in “normal-aging” (SJ4103) worms followed nearly identical trajectories for 4-HNE adduction and methionine oxidation (compare panels Figures 5A,D). However, oxidized Met was markedly and progressively boosted in the presence of Aβ, but deterred by Tau expression (Figure 5D).

### 3.4 AD seed proteins, Aβ and Tau, facilitate precocious aggregation of proteins in insoluble mitochondrial aggregates with age, relative to normal aging

**Figure 6:**
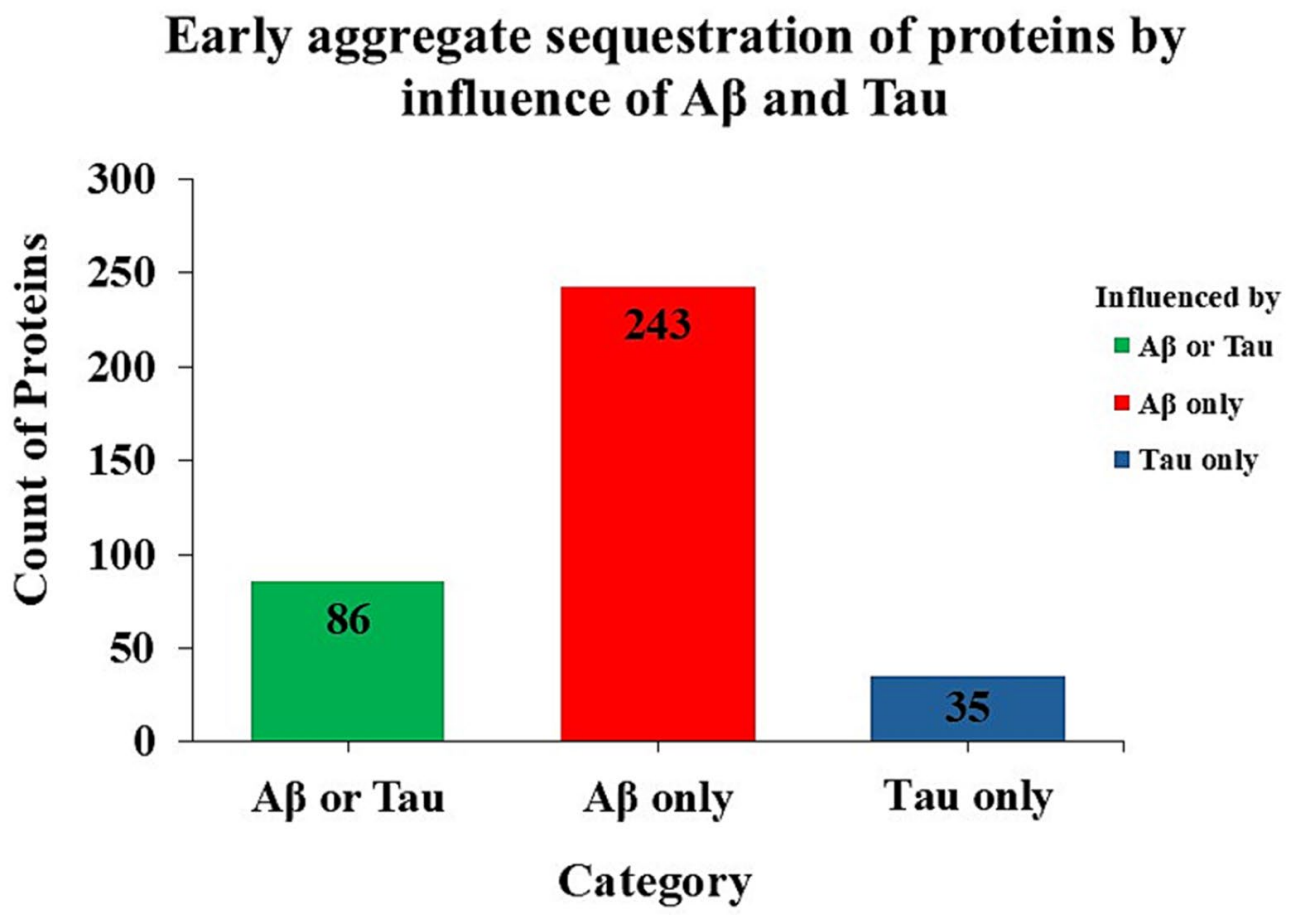
Bar graph summarizing the numbers of proteins recruited into mitochondrial insoluble aggregates due to transgenic expression of Aβ or Tau at the earliest stages of *C. elegans* aging (adult day-1 and day-5). Proteins were included if they showed ≥2-fold elevation in insoluble aggregates compared to age-matched controls without seed protein.

To analyze the impact of Aβ and Tau on the early aggregation propensity of proteins, we focused on the adult day-1 and adult day-5 timepoints, which denote the earliest stages of *C. elegans* aging. To filter the proteins, we used a criterion of at least 2-fold elevation of protein abundance in insoluble aggregates in the presence of Aβ or Tau, compared to normal aging (no seed protein) at the same age. The number of aggregate proteins influenced by Aβ or Tau is depicted in bar graph below (Figure 6). The full list of proteins in insoluble aggregates influenced by Aβ and Tau is provided as Supplementary Table 1.

### 3.5 Aβ promotes early aggregation of metabolic, cytoskeletal, and nucleic acid machinery

**Figure 7:**
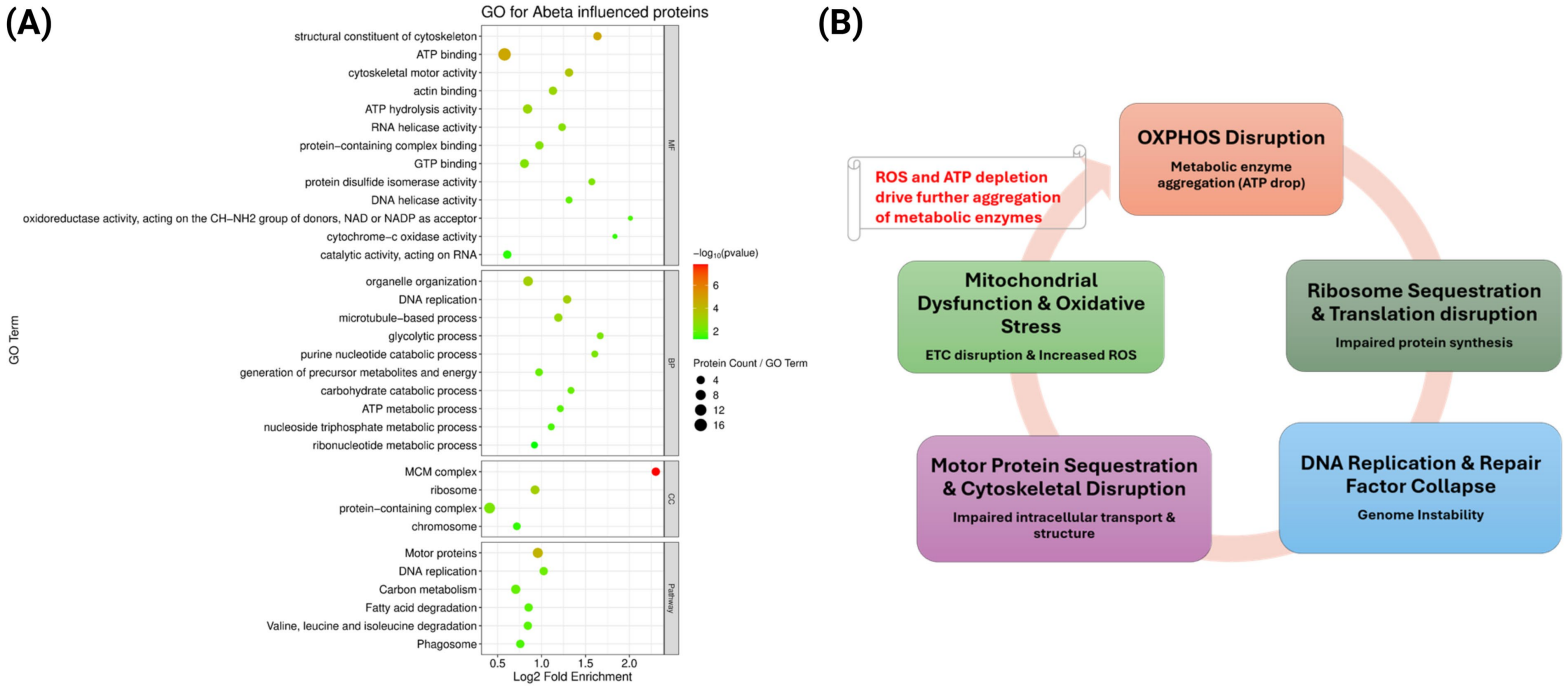
**(A)** Bubble plot representing GO enrichment analysis for Aβ-mediated early aggregating proteins in comparison to normal aging, with bubble size indicating gene count and color representing adjusted *p-*value <0.05 (Benjamini-Hochberg correction). MF, Molecular Function; BP, Biological Processes, CC, Cellular Component. **(B)** Diagram showing the cyclical relationships and interplay between the GO terms in panel (A). This model shows the potential pathway of Aβ-driven proteostasis collapse.

We identified 329 proteins that were induced by Aβ to enter mitochondrial insoluble aggregates, of which 137 showed >5-fold higher abundance relative to normal aging and were selected for enrichment analysis. GO terms revealed overrepresentation of energy metabolism, including ATP metabolic process, glycolysis, carbon metabolism, and cytochrome-c oxidase activity. A second cluster involved cytoskeletal and motor functions, with enrichment for actin binding, cytoskeletal motor activity, and structural constituent of cytoskeleton, pointing to recruitment of actin-associated and motor proteins. A third set mapped to nucleic-acid related processes including RNA helicase activity, DNA helicase activity, and catalytic activity acting on RNA. Strikingly, DNA replication factors such as the MCM complex, DNA replication, and chromosome organization were highly enriched. This set of annotation terms comprising metabolism, cytoskeleton, RNA processing, and replication differs sharply from the profile of age-related aggregates, indicating that Aβ expression promotes early sequestration of proteins essential for energy production, structural integrity, and genome maintenance (Figure 7).

### 3.6 Tau accelerates aggregation of trafficking, vesicular, and nuclear-pore components

**Figure 8:**
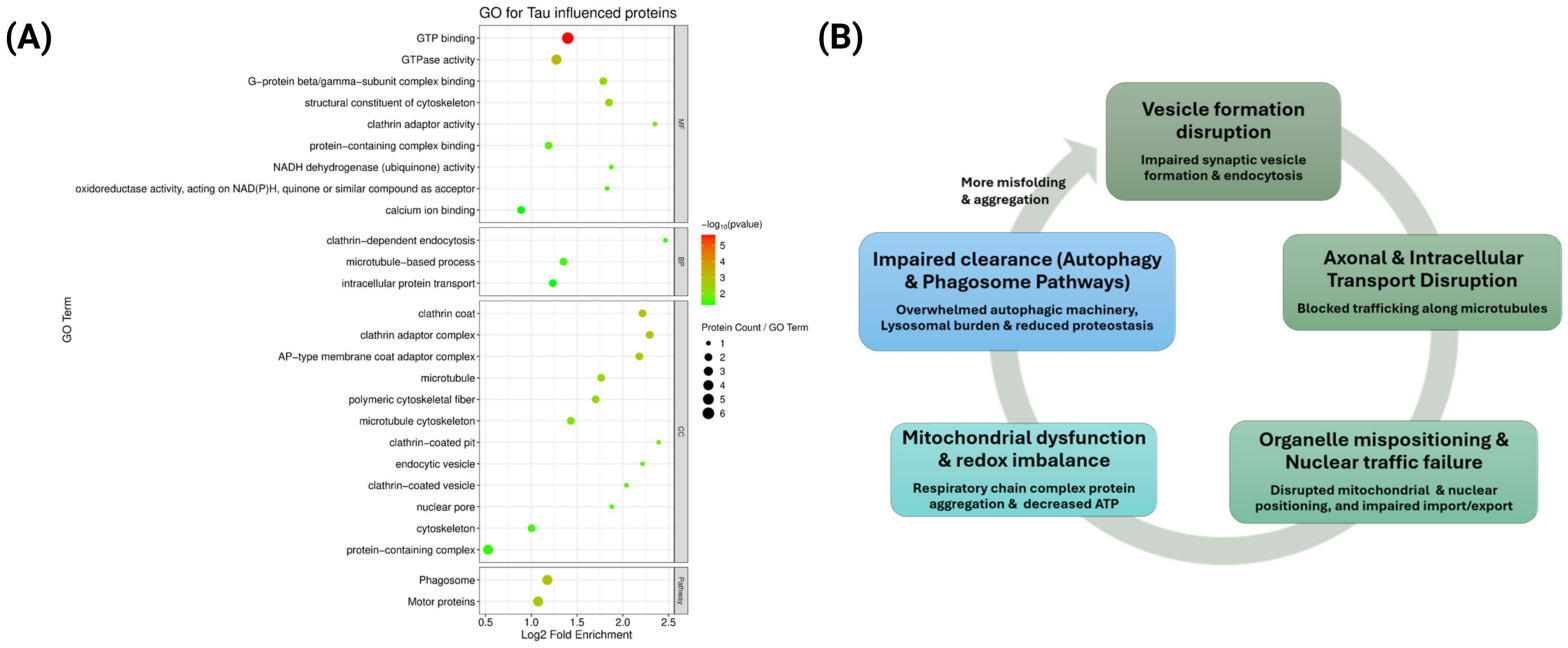
**(A)** Bubble plot representing GO enrichment analysis for Tau-mediated early aggregating proteins in comparison to normal aging, where bubble size indicates gene count and color represents adjusted *p*-value <0.05 (Benjamini-Hochberg correction). MF, Molecular Function; BP, Biological Processes; CC, Cellular Component. **(B)** The diagram represents the cyclical relationships and interplay between the GO terms in panel **(A)**. This model shows the potential pathway of Tau-mediated proteostasis collapse.

Tau favored the early aggregation of 130 proteins, 97 of which had >5-fold higher abundance than in normal-aging nematodes; these 97 Tau-responsive proteins were pursued by GO meta-analysis. Annotation-term enrichment was most significant for vesicle -trafficking and cytoskeletal-system terms, which include GTP binding, GTPase activity, clathrin adaptor activity, and NADH dehydrogenase, implicating signaling, membrane transport, and mitochondrial electron transport chain components. Pathway terms such as clathrin-dependent endocytosis, intracellular protein transport, and microtubule-based processes support this conclusion, with corresponding cellular component annotations also attaining significance, namely clathrin-coated vesicles, adaptor complexes, microtubule cytoskeleton, and endocytic vesicles, marking likely subcellular sites of damage. Additional enrichment for motor-protein and the phagosome-pathway terms points to impaired axonal cargo transport and clearance. Finally, terms including nuclear pore and nuclear pore complex assembly may implicate disruption of nucleo-cytoplasmic transport. Together, these enrichments suggest that Tau accelerates aggregation of proteins vital to vesicle formation, intracellular trafficking, mitochondrial metabolism, and nuclear pore integrity, reflecting an early collapse of cellular logistics and energy homeostasis in the Tauopathy model (Figure 8).

### 3.7 Differential GO enrichments distinguish Aβ- and Tau-driven aggregation

Comparison of GO terms for insoluble proteins favored by Aβ or Tau expression in *C. elegans* models of AD-like aggregation reveals both commonalities and clear distinctions. Both conditions were enriched for motor proteins, consistent with disruption of cytoskeletal transport. Beyond this overlap, however, the profiles diverge. Aβ expression preferentially sequestered nuclear and metabolic factors, with significant enrichment of the MCM complex, ribosomal proteins, and energy-linked enzymes. Terms related to DNA replication, energy metabolism, and ATP-dependent catalysis were uniquely prominent, indicating that Aβ drives early aggregation of factors required for nucleic acid maintenance and bioenergetic capacity. By contrast, Tau favored the trafficking machinery, with enrichment of clathrin-mediated endocytosis, adaptor complexes, nuclear pore components, and cytoskeleton-binding proteins. These annotations point to an early collapse of vesicle cycling, nuclear-cytoplasmic transport, and intracellular clearance pathways.

Taken together, the two seed proteins both provoke premature aggregation relative to normal aging, although via distinct routes: Aβ impairs metabolism, replication, and proteostasis, whereas Tau compromises trafficking and degradation systems. These complementary disruptions highlight parallel yet mechanistically distinct pathways by which AD-linked proteins destabilize the proteome (Table 1).

**Table 1:** Table listing early aggregation signatures of Aβ *vs*. Tau mitochondria in *C. elegans* (Divergent paths to proteostasis failure: Aβ strikes first, Tau completes the collapse).

Feature	Aβ-Induced aggregation	Tau-Induced aggregation
Primary targets	Extracellular interaction partners, metabolic enzymes, and mitochondrial proteins	Vesicle trafficking proteins, motor proteins, and clathrin machinery
Key GO terms (BP)	ATP metabolic process, oxidative phosphorylation, translation initiation	Clathrin-dependent endocytosis, intracellular transport, and microtubule-based processes
Key GO terms (MF)	RNA binding, ATPase activity, NADH dehydrogenase activity	GTP binding, GTPase activity, clathrin adaptor activity, motor protein activity
Key GO terms (CC)	Ribosome, mitochondrial membrane, translation complex	Clathrin coat, microtubule cytoskeleton, endocytic vesicle, adaptor complexes
Impact on proteostasis	Energy depletion → translation arrest → redox imbalance	Transport collapse → vesicle recycling failure → impaired clearance
Functional consequences	Loss of metabolic output and protein synthesis	Synaptic dysfunction and trafficking block
Aggregate composition	Rich in ribosomal proteins, mitochondrial enzymes, and chaperones	Enriched for vesicle coat proteins, motor proteins, and cytoskeletal elements
Clearance burden	Triggers oxidative stress and proteasome overload	Traps proteins involved in the phagosome/autophagy machinery
Converging outcome	Cellular stress amplification and ATP crisis	Collapse of intracellular architecture and transport systems

### 3.8 Cross-species aggregate-proteome overlap unveils conserved AD/aging signatures of mitochondria

**Figure 9:**
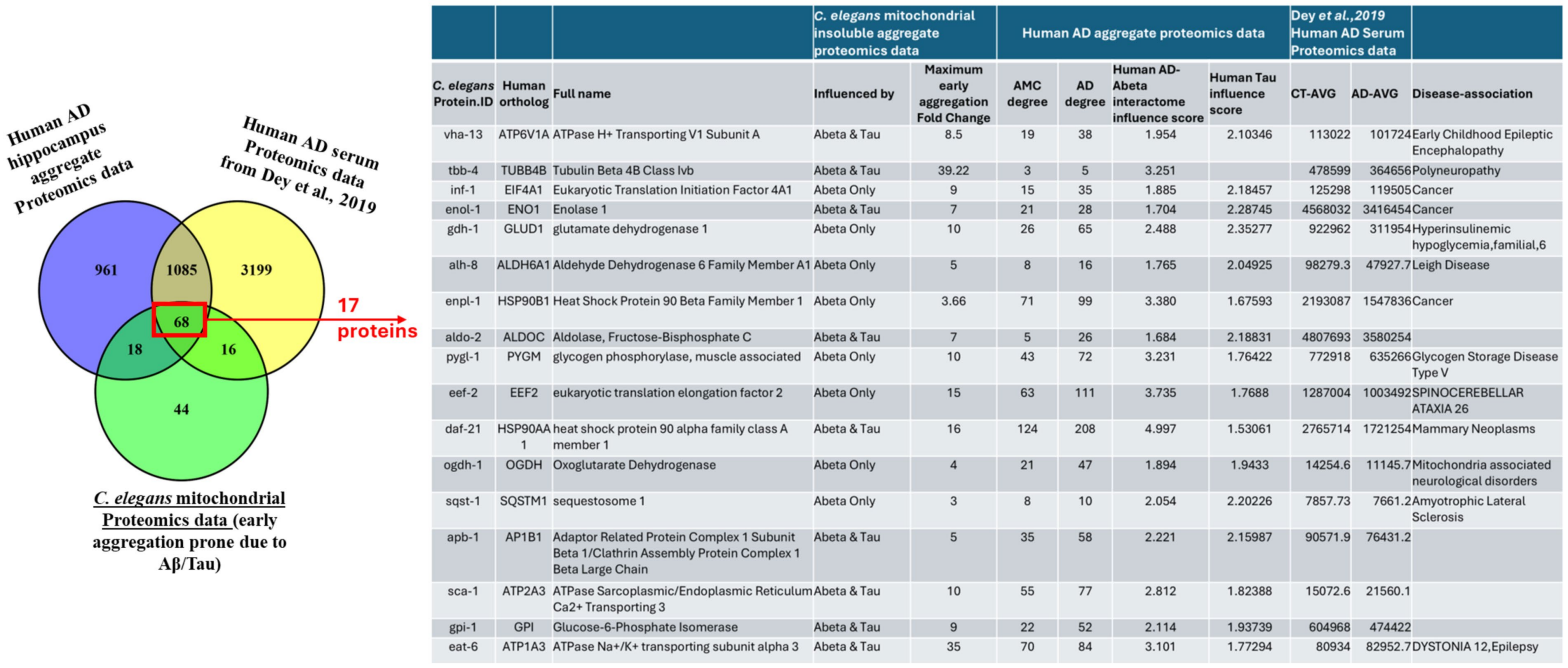
Cross-species overlap between *C. elegans* mitochondrial insoluble aggregates and human Alzheimer disease aggregate proteomes. Venn diagram (left) compares proteins identified in human AD hippocampal aggregate proteomics, human AD serum proteomics (Dey et al., 2019) and *C. elegans* mitochondrial insoluble aggregate proteomics from strains that express Aβ or wild type Tau. The central overlap contains 68 proteins detected in all three datasets, of which 17 early aggregation prone *C. elegans* proteins (boxed and indicated by the red arrow) were prioritized for detailed analysis. The accompanying table (right) lists these 17 proteins, their human orthologs and full names, the aggregation context in *C. elegans* (influenced predominantly by Aβ, Tau or both, with maximum early-aggregate fold change), their network connectivity and Aβ or Tau influence scores in the human AD hippocampal aggregate dataset, relative serum abundance in control and AD cohorts reported by Dey et al. (2019) and representative disease associations drawn from literature.

The concurrence of mitochondrial proteins from *C. elegans* insoluble aggregates with human AD hippocampal aggregate proteins (Balasubramaniam et al., 2019) and serum proteomics data (Dey et al., 2019) yielded 68 common proteins, listed in Supplementary Figure 2. GO and pathway implication of these proteins are summarized in Supplementary Figures 3, 4. For the 68 proteins, we compared human AD hippocampal aggregate interactomes to controls to assess influence scores and network connectivity in the Aβ and Tau interactomes. Based on these rankings we selected the top 17 proteins (Figure 9 and Supplementary Table 2) for further study.

### 3.9 GO enrichment of prioritized proteins implicates energy metabolism and vesicle pathways

GO analysis of the 17 prioritized proteins (restricting FDR to <0.01) revealed a coherent picture in which AD seed proteins preferentially drive sequestration of factors essential for energy metabolism and vesicle biology. The strongest enrichments were for carbohydrate and nucleotide catabolism, including GO terms carbohydrate catabolic process and glycolytic process (GO:0006096; FDR < 2 × 10^−4^), along with a cluster of terms for purine and nucleotide diphosphate catabolism (e.g., GO:0009135, GO:0009205, GO:0009144; each FDR < 2 × 10^−4^). These results indicate that proteins involved in carbohydrate and nucleotide degradation are overrepresented in early-aggregate proteomes.

A second cluster reflected ATP metabolism and enzyme catalysis, with significant enrichment for ATP metabolic process (GO:0046034; FDR < 4 × 10^−4^), ATP hydrolysis activity (GO:0016887), and ATP binding (GO:0005524). These terms implicate sequestration of ATP-dependent enzymes into insoluble aggregates, consistent with impaired energy availability.

The most significant CC annotations were extracellular vesicle and related categories (GO:1903561, GO:0043230, GO:0070062; each FDR < 4 × 10^−9^), implying that many of these proteins are vesicle-associated, although not necessarily vesicular cargo. In KEGG pathways, the top enrichments included carbon metabolism (FDR < 6 × 10^−6^), glycolysis/gluconeogenesis (FDR < 1 × 10^−2^), and necroptosis (FDR < 1 × 10^−2^).

Taken together, these enrichment patterns implicate early sequestration of enzymes involved in glycolysis, the TCA cycle, OXPHOS, and nucleotide metabolism, along with vesicle-mediated processes. This profile underscores an early collapse of cellular energetics and intercellular communication, specific to the presence of AD seed proteins.

### 3.10 Post-translational modifications in the 17 early-aggregating proteins

Across the 17 prioritized proteins, PTM patterns differed sharply among aging, Aβ, and Tau strains, with no indication of a shared, strain-independent modification signature (Table 2). In normal aging (SJ4103), several proteins showed progressive methionine oxidation or phosphorylation across days 1 to 9, most consistently in pygl-1, tbb-4, aldo-2, eat-6, enpl-1, gdh-1, and vha-13, indicating age-linked accumulation of modified sites within mitochondrial insoluble material. By contrast, Aβ-expressing worms (CL2355) showed a far sparser pattern: most proteins lacked detectable PTMs at all surveyed positions, and when present, modifications appeared as isolated late-stage events (e.g., day-9 marks on sqst-1, ogdh-1, sca-1, and HSP90B1), without the progressive age dependence seen in SJ4103. Tau-expressing worms (VH255) exhibited the most restricted profile of all, with only a few single modifications detected at any age, and no protein showing reproducible accumulation across days. Taken together, these results show that PTMs within mitochondrial insoluble aggregates are most abundant and age-progressive in normal aging, sporadic and largely late-appearing under Aβ, and minimal under Tau, indicating that the three conditions generate distinct and non-overlapping modification landscapes rather than a common AD-linked PTM pattern.

**Table 2:** Post-translational modifications identified in the 17 prioritized proteins across mitochondria-enriched insoluble fractions from SJ4103 (aging), CL2355 (Aβ-expressing), and VH255 (Tau-expressing) worms at adult days 1, 5, and 9.

*C. elegans* protein	Human ortholog	PTM site	SJ4103			CL2355			VH255		
			Day1	Day5	Day9	Day1	Day5	Day9	Day1	Day5	Day9
pygl-1	PYGM	M266o		+	+						
		M410o			+						
		M302o		+	+						
		M446o			+						
		S25p				+					
		T26p				+					
sca-1	ATP2A3	S61p							+		
		K503a								+	
		K630a			+						
		K1188a						+			
		T763p						+			
		T767p						+			
		S1120p				−	−	−			
		T112p				−	−	−			
sqst-1	SQSTM1	M442o									
		S669p									
		M370o		+	+						
		T514p				−	−	+			
		S515p				−	−	+			
		S597p	+								
ogdh-1	OGDH	T890p						+			
		S995p						+			
inf-1	EIF4A1	M161o	−		+						
		M174o	+	+	+						
		M183o		+	+						
		M212o			+						
		V321a	−	−	−						
eef-2	EEF2	K414a		+							
tbb-4	TUBB4B	I163a		+							
		M257o	+	+	+						
		M316o			+						
		K379a	+								
		M388o	+	+	+						
aldo-2	ALDOC	T41p	+	−							
		T43p	+	−							
		T122p		+	−						
		R262a	+								
		S40p								+	
alh-8	ALDH6A1	Y433p	+								
eat-6	ATP1A3	T391a		+							
		T611p	−	−	+	−	+	−			
		T613p	−	+	+				−	−	−
		K700a	+		+						
		K700a	+		+						
		M707o	+	+	−						
		S715p	−	+				−			
daf-21	HSP90AA1	T76a		+							
		M698o		+							
enpl-1	HSP90B1	M474o		+	+						
		M518o		+	+						
		K580a				+	+				
		K676a							+		
		T521p					+				
		S681p							+		
gdh-1	GLUD1	S6p	−	+							
		K88a		+							
		K190a	+	+							
		M204o	+								
		S11p						+			
vha-13	ATP6V1A	M23o		+	+						
		M28o		+	+						
		M273o	+	+	+						
		M307o	+	+	+						
		M357o	+	+	+						
		S400p	+	−							
		M533o		+							
		M597o			+						

Sites detected by MS/MS are listed by residue and modification type. A plus sign (+) denotes that the modified peptide was detected; a minus sign (−) indicates the unmodified peptide was detected at that site; and blank cells indicate that the peptide was not detected by PEAKS under the conditions used. Human orthologs are shown for reference.

### 3.11 Behavioral impacts of RNAi knockdowns of 17 early aggregation-prone proteins

**Figure 10:**
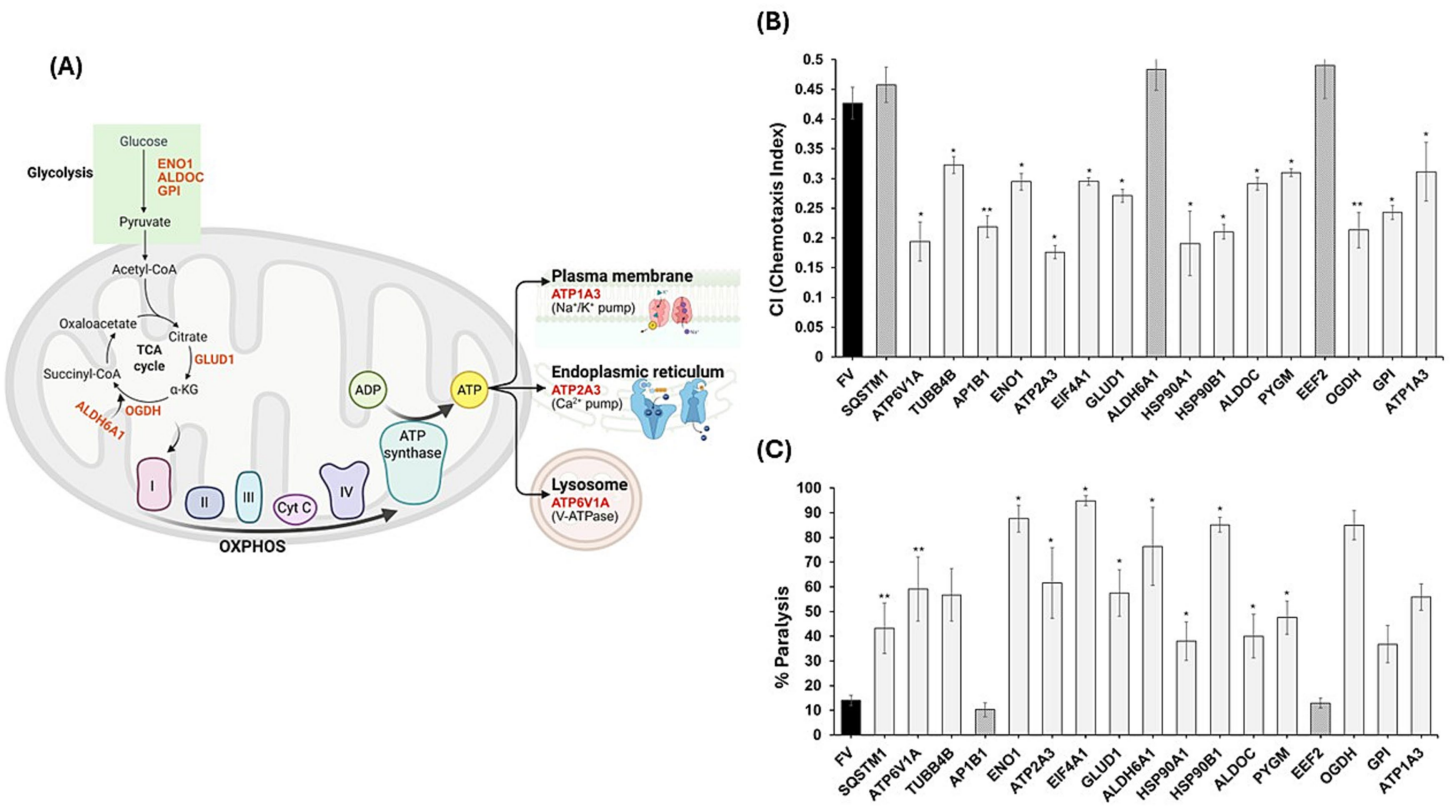
Early aggregation-prone proteins converge on energy metabolism and neuronal functions. **(A)** Prioritized proteins mapped to glycolysis (ENO1, ALDOC, GPI), the TCA cycle (GLUD1, OGDH, ALDH6A1), and ATP-driven pumps/ATPases (ATP1A3, ATP2A3, ATP6V1A), highlighting integration with mitochondrial and ion-transport systems. **(B)** Chemotaxis index (CI) after RNAi knockdown relative to FV. Most knockdowns reduced CI (e.g., AP1B1, OGDH, HSP90B1, TUBB4B, EIF4A1, GLUD1, GPI, ATP6V1A, ALDOC; two-tailed paired *t*, *p* < 0.05). No knockdown significantly increased CI at n = 3; EEF2 and ALDH6A1 showed non-significant upward trends. **(C)** Paralysis under the same conditions. AP1B1 significantly reduced paralysis vs. FV, whereas multiple knockdowns increased paralysis (notably HSP90B1, EIF4A1, ENO1, OGDH, ALDH6A1, ATP1A3, PYGM). Error bars: mean ± SEM across three biological replicates. Significance: *p* < 0.05 (*), *p* < 0.005 (**), two-tailed paired *t*-test.

Seventeen proteins were prioritized for functional tests, based on early aggregation in *C. elegans* Aβ or Tau models and concordant enrichment in human AD insoluble and serum proteomes. RNAi knockdown was assayed in parallel by chemotaxis toward butanol and by age-progressive paralysis (Figure 10).

Across three paired experiments per gene, most RNAi knockdowns impaired behavior relative to FV controls. For chemotaxis, CI decreased significantly for the majority of targets, including AP1B1, OGDH, HSP90B1, TUBB4B, EIF4A1, GLUD1, GPI, ATP6V1A, ALDOC. No knockdown significantly improved CI at n = 3; EEF2 and ALDH6A1 trended higher, whereas SQSTM1 showed a small, non-significant increase. For paralysis, only AP1B1 significantly reduced paralysis compared to FV; several knockdowns increased paralysis substantially; most prominently HSP90B1, EIF4A1, ENO1, OGDH, ALDH6A1, ATP1A3, PYGM. Notably, AP1B1 displayed a trade-off, rescuing paralysis while reducing CI. Together these results indicate that, among tested candidates, behavior is generally worsened by loss of function, with AP1B1 the lone significant paralysis rescue and no significant CI rescues at the current sample size.

## 4 Discussion

We examined the mitochondrial sarkosyl-insoluble proteome across normal aging and two Alzheimer-relevant transgenic perturbations (pan-neuronal Aβ and panneuronal wild-type Tau) and found three central, mechanistically informative results. First, a conserved mitochondrial insoluble core (*n* = 133) links energy metabolism, translation/chaperone machinery and trafficking. The core is substantially represented among insoluble fractions across conditions and ages, and its composition implicates impaired bioenergetics and proteostasis as primary drivers of functional decline. Their aggregation confirms and extends prior evidence that mitochondria represent a central vulnerability in Alzheimer’s disease (AD) and normal aging (Pahal et al., 2025).

Second, pan-neuronal Aβ and pan-neuronal wild-type Tau perturb this program by distinct modalities. Aβ broadly expands the insoluble set and accelerates PTM-linked damage early in adulthood, whereas Tau concentrates the response on trafficking and nuclear-transport machinery and advances timing without equivalent bulk expansion. Third, PTM trajectories (4-HNE adduction, phosphorylation, lysine acetylation and methionine oxidation) mirror these compositional differences and implicate discrete upstream stresses (oxidative/lipid peroxidation versus midlife proteostasis collapse) that converge on mitochondrial dysfunction. Together, these patterns place mitochondrial proteome collapse at the center of aging and AD-seeded pathology, and they argue that the mechanistic routes by which Aβ and Tau produce proteotoxicity are overlapping but distinguishable. Taken together, these data support a working model in which Aβ imposes early oxidative/kinase stress that drives enzyme sequestration and ATP shortfall, whereas Tau primarily impairs vesicular and nuclear transport, secondarily compromising mitochondrial turnover and distribution.

### 4.1 Aβ expands proteome insolubility by early oxidative stress and metabolic compromise

Aβ expression produces the largest expansion of the insoluble mitochondrial proteome (≈950 proteins), with a striking proportion unique to the Aβ condition and with many proteins present as early as adult day-1. This expansion is accompanied by pronounced early PTM burden: Day-1 counts for 4-HNE adducts and STY phosphorylation are substantially elevated in CL2355 relative to controls (4-HNE Day-1: CL2355 = 329 vs. SJ4103 = 201; STY Day-1: CL2355 = 557 vs. SJ4103 = 340). Methionine oxidation on Day-1 is likewise higher under Aβ (CL2355 Day-1 M oxidation = 282 vs. SJ4103 = 208). These signals together implicate early mitochondrial redox stress and lipid peroxidation as drivers of rapid aggregate stabilization and enzyme sequestration. Our observations are consistent with high oxidative burden reported in AD hippocampus (Lovell et al., 1997) and hyperphosphorylated Tau and related proteins contributing to AD pathology (Hanger et al., 2009). Lysine acetylation also peaked early in Aβ (CL2355 Day-1 K acetylation = 217, *vs.* SJ4103 = 134) which is in line with previously reported impaired energy metabolism by aberrant acetylation of mitochondrial enzymes (Choudhary et al., 2009).

Mechanistically, the early sequestration of glycolytic and TCA enzymes (for example, elevated day-1 representation of pyruvate kinases and OGDH in insoluble fractions) links the PTM burden to a plausible metabolic collapse, i.e., loss of soluble enzyme activity will reduce ATP generation and antioxidant capacity, thereby promoting further oxidative PTMs and aggregate persistence. This is consistent with prior reports of pyruvate kinase aggregation and reduced glycolytic flux in aging mammalian tissues (Bie et al., 2024). In short, Aβ appears to initiate a feed-forward loop (Aβ → early oxidative/PTM load → metabolic enzyme sequestration → energetic collapse → more aggregation) that accelerates proteostasis failure. These observations align with reports that mitochondrial stress and hypometabolism precede degeneration in mammalian AD models and human preclinical AD samples (Mosconi et al., 2007). This model predicts reductions in basal and ATP-linked respiration together with increased proton leakage in Day-1 worms, with partial reversal by anti-lipoperoxidation agents or kinase/phosphatase re-balancing. Restoration of soluble glycolytic/ TCA enzyme pools should coincide with improved ATP and decreased oxidative PTMs.

### 4.2 Tau disrupts trafficking and nuclear transport which advances proteostasis failure

By contrast, pan-neuronal wild-type Tau yields a smaller, core-weighted insoluble set (~205 proteins) that attains a Day-5 plateau earlier than normal aging but does not produce the Aβ-scale bulk expansion. Tau-responsive aggregates are enriched for clathrin adaptors, endocytic machinery, motor proteins and a nuclear pore component (Nup98 ortholog), suggesting that Tau’s principal impact is on vesicular traffic, organelle positioning, and nucleo-cytoplasmic transport. All these observations align closely with prior studies showing Tau-mediated disruption of nucleocytoplasmic transport (Eftekharzadeh et al., 2018) and its binding to presynaptic vesicles, impeding vesicular mobility, and disrupting clathrin-mediated endocytosis (Zhou et al., 2017; Yu et al., 2019). The PTM trajectories for Tau more closely resemble accelerated aging by showing midlife peaks (Day-5) in several modification classes, which is consistent with progressive chaperone/clearance failure rather than an immediate lipid-peroxidation assault. This pattern supports a mechanistic model in which soluble Tau species perturb microtubule integrity and trafficking, thereby indirectly compromising mitochondrial distribution, turnover and local bioenergetics.

### 4.3 PTM dynamics refine mechanistic inference

Quantifying four PTM classes across Day-1/5/9 clarifies upstream stress modalities. The Aβ condition’s early elevation of 4-HNE and methionine oxidation together with hyperphosphorylation at Day-1 strongly implicate immediate oxidative lipid damage and kinase/phosphatase imbalance as accelerants of aggregation (Day-1 HNE: CL2355 = 329; Day-1 methionine oxidation: CL2355 = 282; Day-1 phosphorylation: CL2355 = 557). Normal aging and Tau show midlife (Day-5) maxima in several PTM classes, indicating a slower, proteostasis-collapse trajectory dependent on chaperone exhaustion and clearance failure. Acetylation patterns (lysine-acetyl counts) showed similar divergence, with Aβ producing higher early acetylation burdens than either aging or Tau. Overall, PTM trajectories are not incidental: they are coherent with compositional shifts and provide testable hypotheses about the enzymes (acetyltransferases/deacetylases, kinases/phosphatases) and redox pathways that set aggregate fate. We anticipate early defects in endocytosis/axonal transport and nuclear import–export, with mitochondrial consequences emerging later (altered distribution, mitophagy flux, and ΔΨm). Stabilizing microtubule transport or nuclear-pore function should attenuate the Day-5 PTM peaks and normalize markers of trafficking.

### 4.4 Cross-species overlap and functional significance

Cross-species overlap with human AD insoluble aggregate proteomes (Ayyadevara et al., 2016a) and serum proteome (Dey et al., 2019) returned 68 shared proteins, from which 17 were prioritized by network metrics and conservation. Functional perturbation by RNAi of most of these 17 candidates worsened worm phenotypes (paralysis or chemotaxis deficits), confirming that several aggregate-enriched proteins are not neutral bystanders but are functionally required. The concordance among proteomic enrichment, PTM signatures, and behavioral deterioration strengthens a causal interpretation that sequestration and/or modification of core metabolic and trafficking proteins can drive organismal dysfunction. These findings rationalize prioritizing this compact set for biochemical assays and translational biomarker testing. Independent human and mammalian studies also reported early changes in some glycolytic/TCA enzymes and chaperones in AD, reinforcing these proteins as biomarkers (Reis-Rodrigues et al., 2012; de Geus et al., 2023; Stempler et al., 2014; Martin et al., 2008).

Our results are consistent with prior work showing age-dependent accrual of aggregation-prone metabolic and proteostasis proteins (Ayyadevara et al., 2015) and with mammalian observations of early brain hypometabolism in preclinical AD and Tau-linked disruption of nuclear-pore components (Eftekharzadeh et al., 2018; Diez et al., 2022). The present *C. elegans* data extend these mammalian findings by isolating PTM trajectories and by showing that Aβ and Tau drive divergent, yet convergent, routes to mitochondrial proteome collapse. Consistent with the proteome-level differences, PTM mapping across the same 17 candidates showed abundant, age-progressive modifications during normal aging, scattered late-stage changes under Aβ influence, and minimal detectable PTMs in the presence of Tau, strengthening the impression that these AD-mimicking conditions impose distinct and non-overlapping biochemical stresses on the mitochondrial proteome. Limitations and planned validations including respiratory flux assays at Day-1/Day-5 and transport readouts are summarized below.

### 4.5 Pharmacologic strategies

Therapeutic strategies should be tailored to these divergent routes. Against Aβ-dominated pathology, approaches that limit lipid peroxidation (anti-HNE strategies), preserve mitochondrial enzyme activity, or stabilize metabolic enzyme solubility may protect energetic capacity and break the feed-forward aggregation loop. Against Tau-dominant pathology, interventions that enhance vesicle trafficking, preserve microtubule integrity, or restore nuclear-cytoplasmic transport may better prevent focal proteostasis collapse. The 17 prioritized proteins offer immediate candidate biomarkers (altered abundance, insoluble fragments, or PTM states) that could be assayed in CSF/plasma and tested for early predictive value.

## 5 Limitations

While *C. elegans* captures conserved features of proteostasis, it cannot model full mammalian neuronal complexity or cell-type diversity. Respiratory flux, ΔΨm, ROS, ATP, and PTM stoichiometry were not measured *in vivo* in this study. PTM counts reflect identification frequency, not stoichiometry, and may be biased by peptide detectability. Cross-species overlaps, though striking, remain inferential until confirmed in human biofluids or tissues. Finally, RNAi knockdowns demonstrate loss-of-function consequences but cannot separate effects of sequestration from irreversible chemical modification; complementary biochemical assays will be needed.

## 6 Future directions

The observations presented here could be pursued by Seahorse mitochondrial-stress assays on days 1, 5, and 9, in Aβ- and Tau-expressing strains, along with ΔΨm/ROS/ATP measurements, to test predicted energy deficits and proton leakage. This may entail measuring activities of key metabolic enzymes in isolated mitochondria from worm and mammalian models to assess whether antioxidant/anti-HNE strategies help to preserve activity and prevent aggregation. In the Tau-expressing strain, we propose to assess endocytosis and axonal transport (FM-dye uptake, cargo tracking) and nuclear import–export (NLS/NES reporters), and to quantify mitophagic flux and mitochondrial distribution. Orthologs of the 17 prioritized proteins, and their PTM status, should be evaluated in rodent AD models and in human CSF/plasma cohorts as biomarker candidates. Finally, because Aβ and Tau act via different mechanisms, interventions will be tailored to the targeted route: antioxidant and metabolic stabilizers for Aβ-facilitated pathology; trafficking/ transport support and clearance enhancers for Tauopathy.

## Data Availability

The original contributions presented in the study are included in the article/supplementary material, further inquiries can be directed to the corresponding authors.

## References

[ref1] Aufschnaiter A. Kohler V. Diessl J. Peselj C. Carmona-Gutierrez D. Keller W. . (2016). Mitochondrial lipids in neurodegeneration. Cell Tissue Res. 367, 125–140. doi: 10.1007/s00441-016-2463-1, 27449929 PMC5203858

[ref2] Ayyadevara S. Balasubramaniam M. Gao Y. Yu L. R. Alla R. Shmookler Reis R. J. (2015). Proteins in aggregates functionally impact multiple neurodegenerative disease models by forming proteasome-blocking complexes. Aging Cell 14, 35–48. doi: 10.1111/acel.12296, 25510159 PMC4326912

[ref3] Ayyadevara S. Balasubramaniam M. Parcon P. A. Barger S. W. Griffin W. S. T. Alla R. . (2016a). Proteins that mediate protein aggregation and cytotoxicity distinguish Alzheimer's hippocampus from normal controls. Aging Cell 15, 924–939. doi: 10.1111/acel.12501, 27448508 PMC5013017

[ref4] Ayyadevara S. Balasubramaniam M. Suri P. Mackintosh S. G. Tackett A. J. Sullivan D. H. . (2016b). Proteins that accumulate with age in human skeletal-muscle aggregates contribute to declines in muscle mass and function in *Caenorhabditis elegans*. Aging 8, 3486–3497. doi: 10.18632/aging.101141, 27992858 PMC5270681

[ref5] Balasubramaniam M. Ayyadevara S. Ganne A. Kakraba S. Penthala N. R. Du X. . (2019). Aggregate interactome based on protein cross-linking interfaces predicts drug targets to limit aggregation in neurodegenerative diseases. iScience. 20:31593839, 248–264. doi: 10.1016/j.isci.2019.09.026 PMC6817627 31593839

[ref6] Balasubramaniam M. Ganne A. Mainali N. Pahal S. Ayyadevara S. Shmookler Reis R. J. (2023). Alzheimer’s-specific brain amyloid interactome: neural-network analysis of intra-aggregate crosslinking identifies novel drug targets. iScience. 27:108745. doi: 10.1016/j.isci.2023.108745 38274404 PMC10809092

[ref7] Bar-Ziv R. Bolas T. Dillin A. (2020a). Systemic effects of mitochondrial stress. EMBO Rep. 21:e50094. doi: 10.15252/embr.202050094, 32449292 PMC7271648

[ref8] Bar-Ziv R. Bolas T. Dillin A. (2020). Systemic effects of mitochondrial stress. EMBO Rep. 21:e50094. doi: 10.15252/embr.202050094 32449292 PMC7271648

[ref9] Bender T. Lewrenz I. Franken S. Baitzel C. Voos W. (2011). Mitochondrial enzymes are protected from stress-induced aggregation by mitochondrial chaperones and the Pim1/LON protease. Mol. Biol. Cell 22, 541–554. doi: 10.1091/mbc.e10-08-0718, 21209324 PMC3046053

[ref10] Bie J. Li R. Li Y. Song C. Chen Z. Zhang T. . (2024). PKM2 aggregation drives metabolism reprograming during aging process. Nat. Commun. 15:5761. doi: 10.1038/s41467-024-50242-y, 38982055 PMC11233639

[ref11] Bonora M. Patergnani S. Ramaccini D. Morciano G. Pedriali G. Kahsay A. . (2020). Physiopathology of the permeability transition pore: molecular mechanisms in human pathology. Biomolecules 10:10. doi: 10.3390/biom10070998, 32635556 PMC7408088

[ref12] Bratić A. M. Larsson N.-G. (2013). The role of mitochondria in aging. J. Clin. Invest. 123, 951–957. doi: 10.1172/JCI64125, 23454757 PMC3582127

[ref13] Cai Q. Tammineni P. (2016). Alterations in mitochondrial quality control in Alzheimer’s disease. Front. Cell. Neurosci. 10:24. doi: 10.3389/fncel.2016.00024, 26903809 PMC4746252

[ref14] Castro I. P. Costa A. C. Lam D. Tufi R. Fedele V. Moisoi N. . (2012). Genetic analysis of mitochondrial protein misfolding in *Drosophila melanogaster*. Cell Death Differ. 19, 1308–1316. doi: 10.1038/cdd.2012.5 22301916 PMC3392634

[ref15] Chistiakov D. A. Sobenin I. A. Revin V. V. Orekhov A. N. Bobryshev Y. V. (2014). Mitochondrial aging and age-related dysfunction of mitochondria. Biomed. Res. Int. 2014, 1–7. doi: 10.1155/2014/238463, 24818134 PMC4003832

[ref16] Choudhary C. Kumar C. Gnad F. Nielsen M. L. Rehman M. Walther T. C. . (2009). Lysine acetylation targets protein complexes and co-regulates major cellular functions. Science 325, 834–840. doi: 10.1126/science.1175371, 19608861

[ref17] Cilleros-Holgado P. Gómez-Fernández D. Piñero-Pérez R. Romero-Domínguez J. M. Reche-López D. López-Cabrera A. . (2023). Mitochondrial quality control via mitochondrial unfolded protein response (mtUPR) in ageing and neurodegenerative diseases. Biomolecules 13:13. doi: 10.3390/biom13121789, 38136659 PMC10741690

[ref18] Cuanalo-Contreras K. Moreno-Gonzalez I. (2019). Natural products as modulators of the Proteostasis machinery: implications in neurodegenerative diseases. Int. J. Mol. Sci. 20:20. doi: 10.3390/ijms20194666, 31547084 PMC6801507

[ref19] Cuanalo-Contreras K. Schulz J. Mukherjee A. Park K.-W. Armijo E. Soto C. (2023). Extensive accumulation of misfolded protein aggregates during natural aging and senescence. Front. Aging Neurosci. 14:1090109. doi: 10.3389/fnagi.2022.1090109, 36778589 PMC9909609

[ref20] D’alessandro M. C. B. Kanaan S. Geller M. Praticò D. Daher J. P. L. (2025). Mitochondrial dysfunction in Alzheimer’s disease. Ageing Res. Rev. 107:102713. doi: 10.1016/j.arr.2025.102713, 40023293

[ref21] de Geus M. B. Leslie S. N. Lam T. Wang W. Roux-Dalvai F. Droit A. . (2023). Mass spectrometry in cerebrospinal fluid uncovers association of glycolysis biomarkers with Alzheimer’s disease in a large clinical sample. Sci. Rep. 13:22406. doi: 10.1038/s41598-023-49440-3, 38104170 PMC10725469

[ref22] Devi L. Raghavendran V. Prabhu B. M. Avadhani N. G. Anandatheerthavarada H. K. (2008). Mitochondrial import and accumulation of α-synuclein impair complex I in human dopaminergic neuronal cultures and Parkinson disease brain*. J. Biol. Chem. 283, 9089–9100. doi: 10.1074/jbc.M710012200, 18245082 PMC2431021

[ref23] Dey K. K. Wang H. Niu M. Bai B. Wang X. Li Y. . (2019). Deep undepleted human serum proteome profiling toward biomarker discovery for Alzheimer’s disease. Clin. Proteomics 16:16. doi: 10.1186/s12014-019-9237-1, 31019427 PMC6472024

[ref24] Diez L. Kapinos L. E. Hochmair J. Huebschmann S. Dominguez-Baquero A. Vogt A. . (2022). Phosphorylation but not oligomerization drives the accumulation of Tau with nucleoporin Nup98. Int. J. Mol. Sci. 23:3495. doi: 10.3390/ijms23073495, 35408855 PMC8998617

[ref25] Eftekharzadeh B. Daigle J. G. Kapinos L. E. Coyne A. Schiantarelli J. Carlomagno Y. . (2018). Tau protein disrupts nucleocytoplasmic transport in Alzheimer’s disease. Neuron 99, 925–940.e7. e7. doi: 10.1016/j.neuron.2018.07.039, 30189209 PMC6240334

[ref26] Esselun C. Theyssen E. Eckert G. P. (2021). Effects of urolithin a on mitochondrial parameters in a cellular model of early Alzheimer disease. Int. J. Mol. Sci. 22:333. doi: 10.3390/ijms22158333, 34361099 PMC8347929

[ref27] Gudenschwager C. Chavez I. Cárdenas C. González-Billault C. (2021). Directly reprogrammed human neurons to understand age-related energy metabolism impairment and mitochondrial dysfunction in healthy aging and neurodegeneration. Oxidative Med. Cell. Longev. 2021:52. doi: 10.1155/2021/5586052, 34950417 PMC8691983

[ref28] Hanger D. P. Anderton B. H. Noble W. (2009). Tau phosphorylation: the therapeutic challenge for neurodegenerative disease. Trends Mol. Med. 15, 112–119. doi: 10.1016/j.molmed.2009.01.003, 19246243

[ref29] Hoppe T. Cohen E. (2020). Organismal Protein Homeostasis Mechanisms. Genetics 215, 889–901. doi: 10.1534/genetics.120.301283, 32759342 PMC7404231

[ref30] Ingram T. L. Chakrabarti L. (2016). Proteomic profiling of mitochondria: what does it tell us about the ageing brain? Aging 8, 3161–3179. doi: 10.18632/aging.101131, 27992860 PMC5270661

[ref31] Jin M. Cai S.-Q. (2022). Mechanisms underlying brain aging under Normal and pathological conditions. Neurosci. Bull. 39, 303–314. doi: 10.1007/s12264-022-00969-9, 36437436 PMC9905409

[ref32] Kalia S. K. Kalia L. V. Kalia L. V. McLean P. J. (2010). Molecular chaperones as rational drug targets for Parkinson's disease therapeutics. CNS Neurol. Disord. Drug Targets 9, 741–753. doi: 10.2174/187152710793237386, 20942788 PMC3364514

[ref33] Köhler F. Müller-Rischart A. K. Conradt B. Rolland S. G. (2015). The loss of LRPPRC function induces the mitochondrial unfolded protein response. Aging 7, 701–712. doi: 10.18632/aging.100812, 26412102 PMC4600627

[ref34] Kolberg L. Raudvere U. Kuzmin I. Adler P. Vilo J. Peterson H. (2023). G: profiler—interoperable web service for functional enrichment analysis and gene identifier mapping (2023 update). Nucleic Acids Res. 51, W207–W212. doi: 10.1093/nar/gkad347, 37144459 PMC10320099

[ref35] Krämer L. Groh C. Herrmann J. M. (2020). The proteasome: friend and foe of mitochondrial biogenesis. FEBS Lett. 595, 1223–1238. doi: 10.1002/1873-3468.14010, 33249599

[ref36] Liu J. Lillo C. Jonsson P. A. Vande Velde C. Ward C. M. Miller T. M. . (2004). Toxicity of familial ALS-linked SOD1 mutants from selective recruitment to spinal mitochondria. Neuron 43, 5–17. doi: 10.1016/j.neuron.2004.06.016, 15233913

[ref37] Lovell M. Ehmann W. Mattson M. Markesbery W. (1997). Elevated 4-hydroxynonenal in ventricular fluid in Alzheimer’s disease. Neurobiol. Aging 18, 457–461. doi: 10.1016/S0197-4580(97)00108-5, 9390770

[ref38] Martin B. Brenneman R. Becker K. G. Gucek M. Cole R. N. Maudsley S. (2008). iTRAQ analysis of complex proteome alterations in 3xTgAD Alzheimer's mice: understanding the interface between physiology and disease. PLoS One 3:e2750. doi: 10.1371/journal.pone.0002750, 18648646 PMC2453232

[ref39] Moehle E. A. Shen K. Dillin A. (2018). Mitochondrial proteostasis in the context of cellular and organismal health and aging. J. Biol. Chem. 294, 5396–5407. doi: 10.1074/jbc.TM117.000893, 29622680 PMC6462515

[ref40] Morley J. F. Brignull H. R. Weyers J. J. Morimoto R. I. (2002). The threshold for polyglutamine-expansion protein aggregation and cellular toxicity is dynamic and influenced by aging in *Caenorhabditis elegans*. Proc. Natl. Acad. Sci. 99, 10417–10422. doi: 10.1073/pnas.152161099, 12122205 PMC124929

[ref41] Mosconi L. Brys M. Switalski R. Mistur R. Glodzik L. Pirraglia E. . (2007). Maternal family history of Alzheimer's disease predisposes to reduced brain glucose metabolism. Proc. Natl. Acad. Sci. 104, 19067–19072. doi: 10.1073/pnas.0705036104, 18003925 PMC2141909

[ref42] Olesen M. A. Torres A. K. Jara C. Murphy M. P. Tapia-Rojas C. (2020). Premature synaptic mitochondrial dysfunction in the hippocampus during aging contributes to memory loss. Redox Biol. 34:101558. doi: 10.1016/j.redox.2020.101558, 32447261 PMC7248293

[ref43] Pahal S. Mainali N. Balasubramaniam M. Reis R. J. S. Ayyadevara S. (2025). Mitochondria in aging and age-associated diseases. Mitochondrion 82:102022. doi: 10.1016/j.mito.2025.102022, 40023438

[ref44] Pasinelli P. Belford M. E. Lennon N. Bacskai B. J. Hyman B. T. Trotti D. . (2004). Amyotrophic lateral sclerosis-associated SOD1 mutant proteins bind and aggregate with Bcl-2 in spinal cord mitochondria. Neuron 43, 19–30. doi: 10.1016/j.neuron.2004.06.021, 15233914

[ref45] Rai M. Curley M. Coleman Z. Demontis F. (2022). Contribution of proteases to the hallmarks of aging and to age-related neurodegeneration. Aging Cell 21:e13603. doi: 10.1111/acel.13603, 35349763 PMC9124314

[ref46] Reis-Rodrigues P. Czerwieniec G. A. Peters T. W. Evani U. S. Alavez S. Gaman E. A. . (2012). Proteomic analysis of age-dependent changes in protein solubility identifies genes that modulate lifespan. Aging Cell 11, 120–127. doi: 10.1111/j.1474-9726.2011.00765.x, 22103665 PMC3437485

[ref47] Ross J. M. Olson L. Coppotelli G. (2015). Mitochondrial and ubiquitin proteasome system dysfunction in ageing and disease: two sides of the same coin? Int. J. Mol. Sci. 16, 19458–19476. doi: 10.3390/ijms160819458, 26287188 PMC4581307

[ref48] Spillantini M. G. Crowther A. Jakes R. Hasegawa M. Goedert M. (1998). α-Synuclein in filamentous inclusions of Lewy bodies from Parkinson’s disease and dementia with Lewy bodies. Proc. Natl. Acad. Sci. USA 95, 6469–6473. doi: 10.1073/pnas.95.11.6469, 9600990 PMC27806

[ref49] Stempler S. Yizhak K. Ruppin E. (2014). Integrating transcriptomics with metabolic modeling predicts biomarkers and drug targets for Alzheimer's disease. PLoS One 9:e105383. doi: 10.1371/journal.pone.0105383, 25127241 PMC4134302

[ref50] Sukhorukov V. L. Voronkov D. N. Baranich T. I. Mudzhiri N. M. Magnaeva A. S. Illarioshkin S. N. (2021). Impaired mitophagy in neurons and glial cells during aging and age-related disorders. Int. J. Mol. Sci. 22:251. doi: 10.3390/ijms221910251, 34638589 PMC8508639

[ref51] Sutherland T. C. Sefiani A. Horvat D. Huntington T. E. Lei Y. West A. P. . (2021). Age-dependent decline in neuron growth potential and mitochondria functions in cortical neurons. Cells 10:10. doi: 10.3390/cells10071625, 34209640 PMC8306398

[ref52] Swerdlow R. H. (2017). Mitochondria and mitochondrial cascades in Alzheimer’s disease. J Alzheimer's Dis 62, 1403–1416. doi: 10.3233/JAD-170585, 29036828 PMC5869994

[ref53] Tang D. Chen M. Huang X. Zhang G. Zeng L. Zhang G. . (2023). SRplot: a free online platform for data visualization and graphing. PLoS One 18:e0294236. doi: 10.1371/journal.pone.0294236, 37943830 PMC10635526

[ref54] Varghese N. Werner S. Grimm A. Eckert A. (2020). Dietary mitophagy enhancer: a strategy for healthy brain aging? Antioxidants 9:9. doi: 10.3390/antiox9100932, 33003315 PMC7600282

[ref55] Weinert B. T. Timiras P. S. (2003). Invited review: theories of aging. J. Appl. Physiol. 95, 1706–1716. doi: 10.1152/japplphysiol.00288.2003, 12970376

[ref56] Yu A. Fox S. G. Cavallini A. Kerridge C. O’Neill M. J. Wolak J. . (2019). Tau protein aggregates inhibit the protein-folding and vesicular trafficking arms of the cellular proteostasis network. J. Biol. Chem. 294, 7917–7930. doi: 10.1074/jbc.RA119.007527, 30936201 PMC6514629

[ref57] Zhou L. McInnes J. Wierda K. Holt M. Herrmann A. G. Jackson R. J. . (2017). Tau association with synaptic vesicles causes presynaptic dysfunction. Nat. Commun. 8:15295. doi: 10.1038/ncomms15295, 28492240 PMC5437271

