# Supplementary figures and images for: The aggregate proteome of Caenorhabditis elegans mitochondria implicates shared mechanisms of aging and Alzheimer’s disease
Source: Front Aging Neurosci. 2026 Jan 13;17:1713391. doi: 10.3389/fnagi.2025.1713391 (PMC12835372; doi:10.3389/fnagi.2025.1713391)

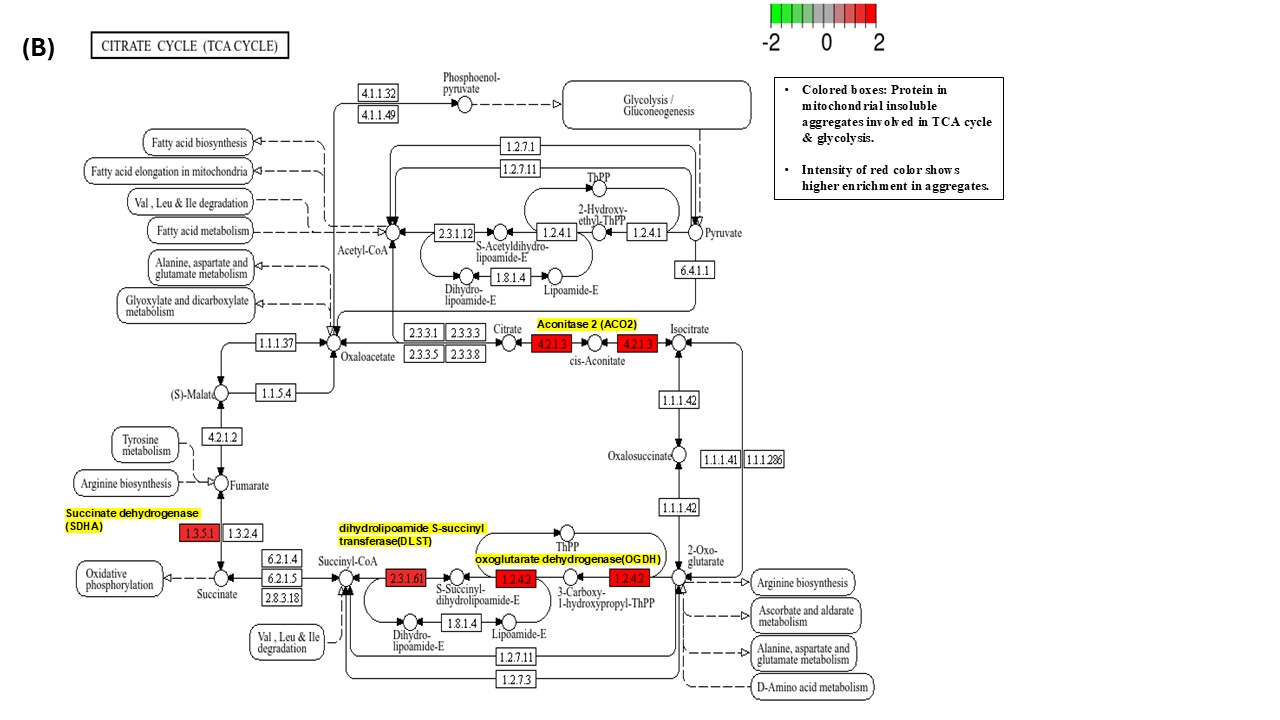

Supplement: Supplementary file 4 [file Image_1.jpeg]

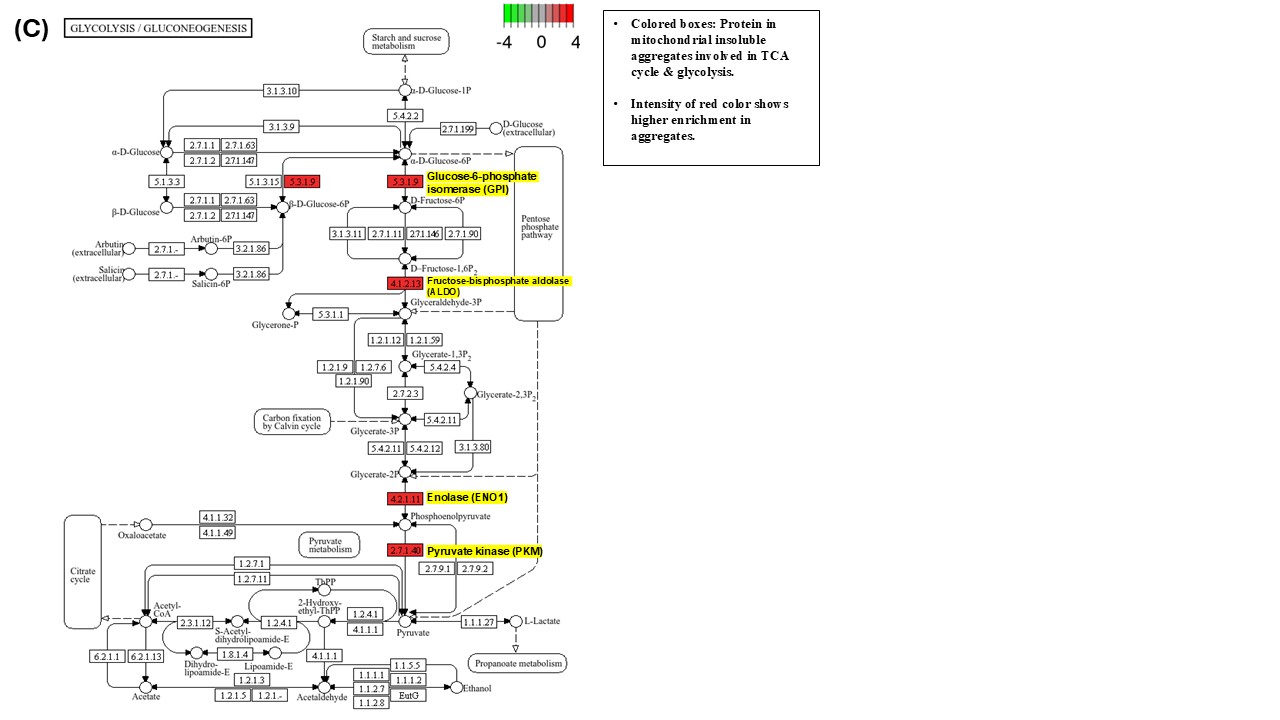

Supplement: Supplementary file 5 [file Image_2.jpeg]

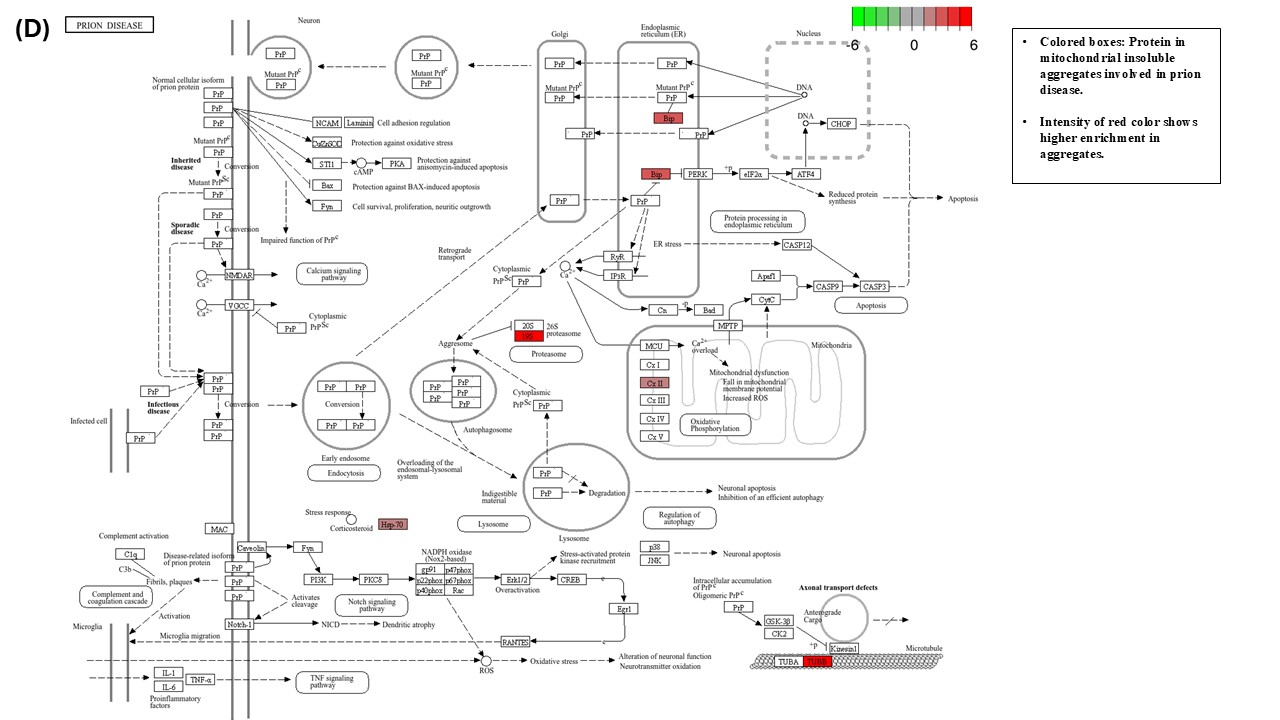

Supplement: Supplementary file 6 [file Image_3.jpeg]

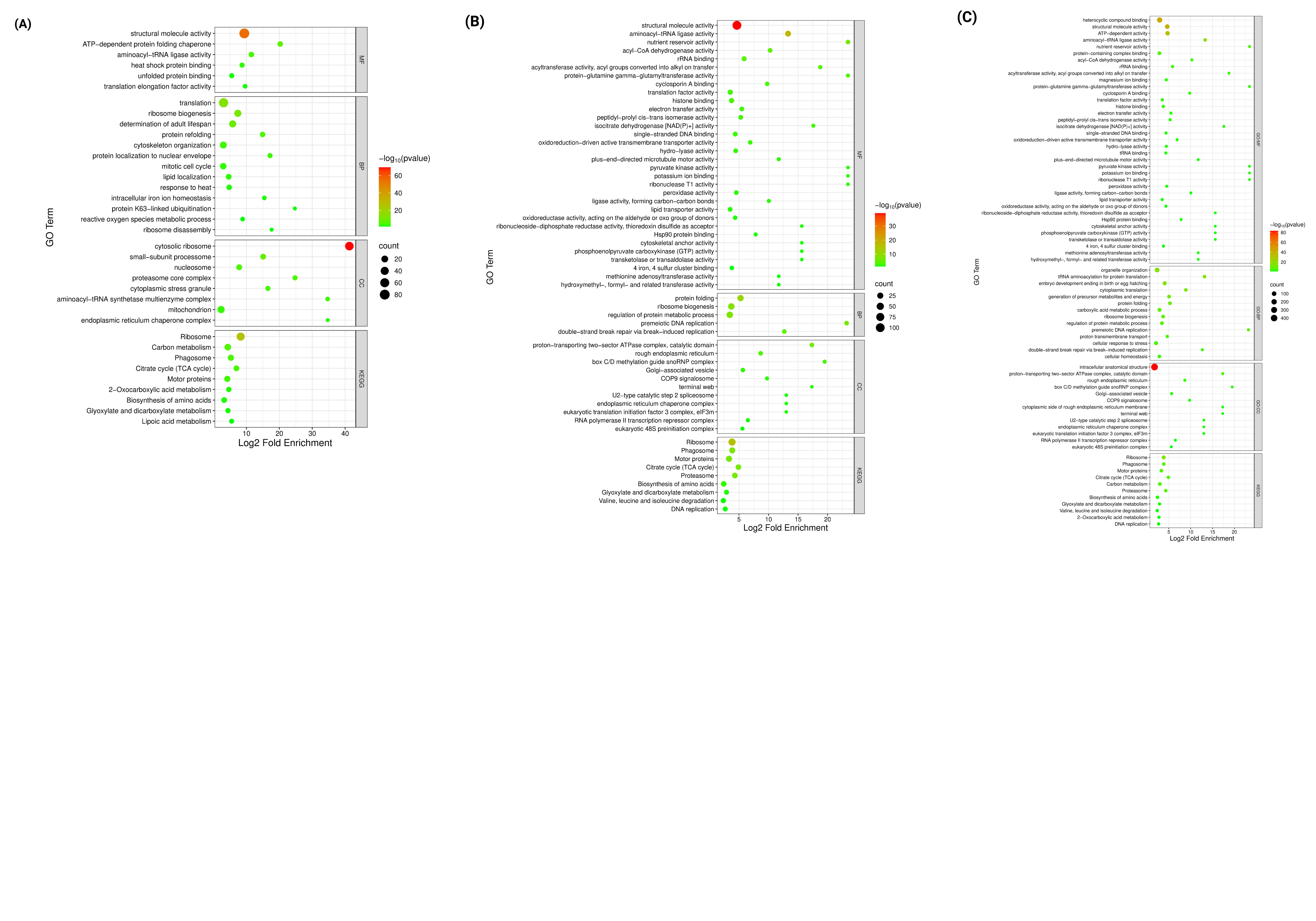

Supplement: Supplementary file 7 [file Image_4.jpeg]

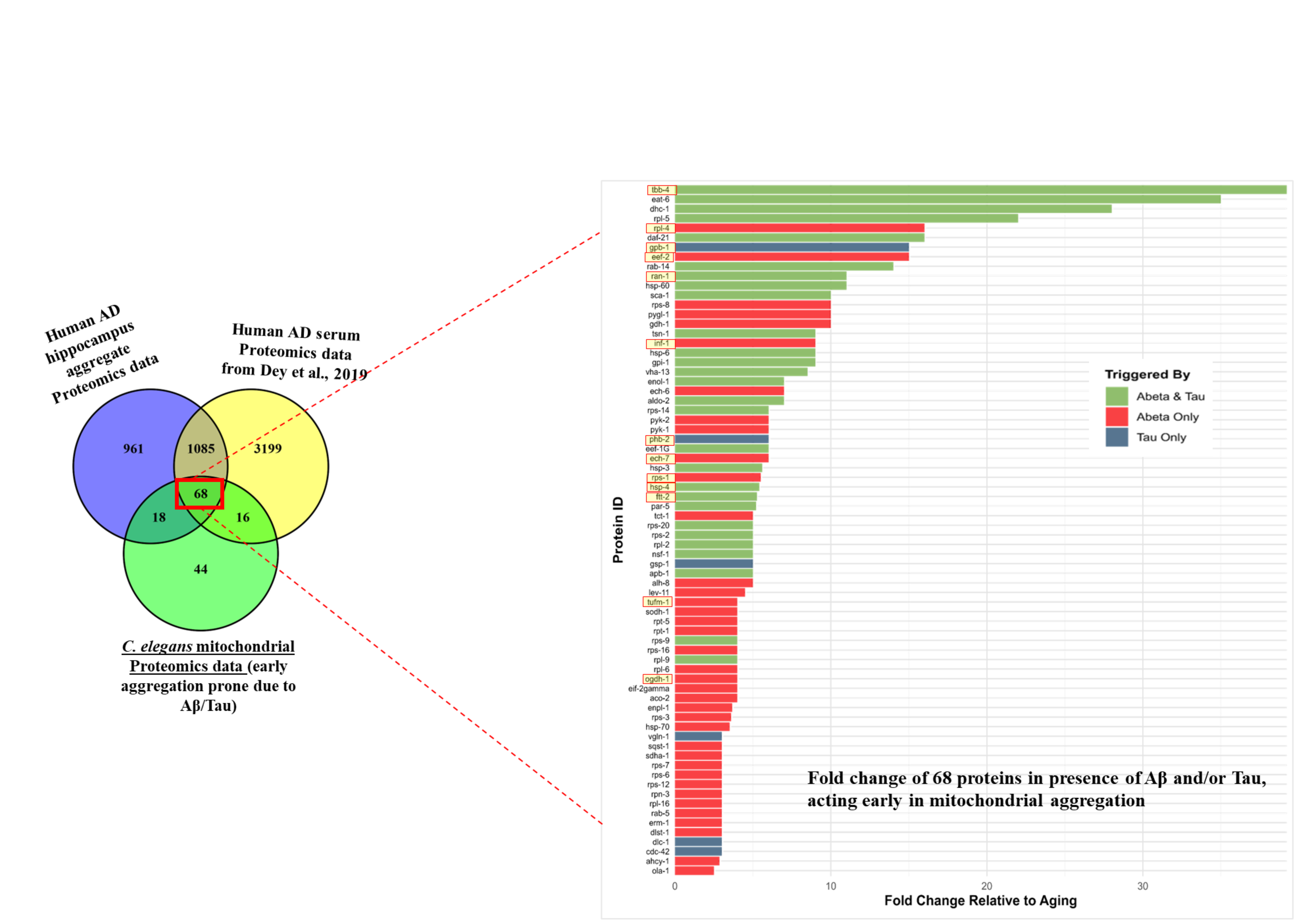

Supplement: Supplementary file 8 [file Image_5.png]

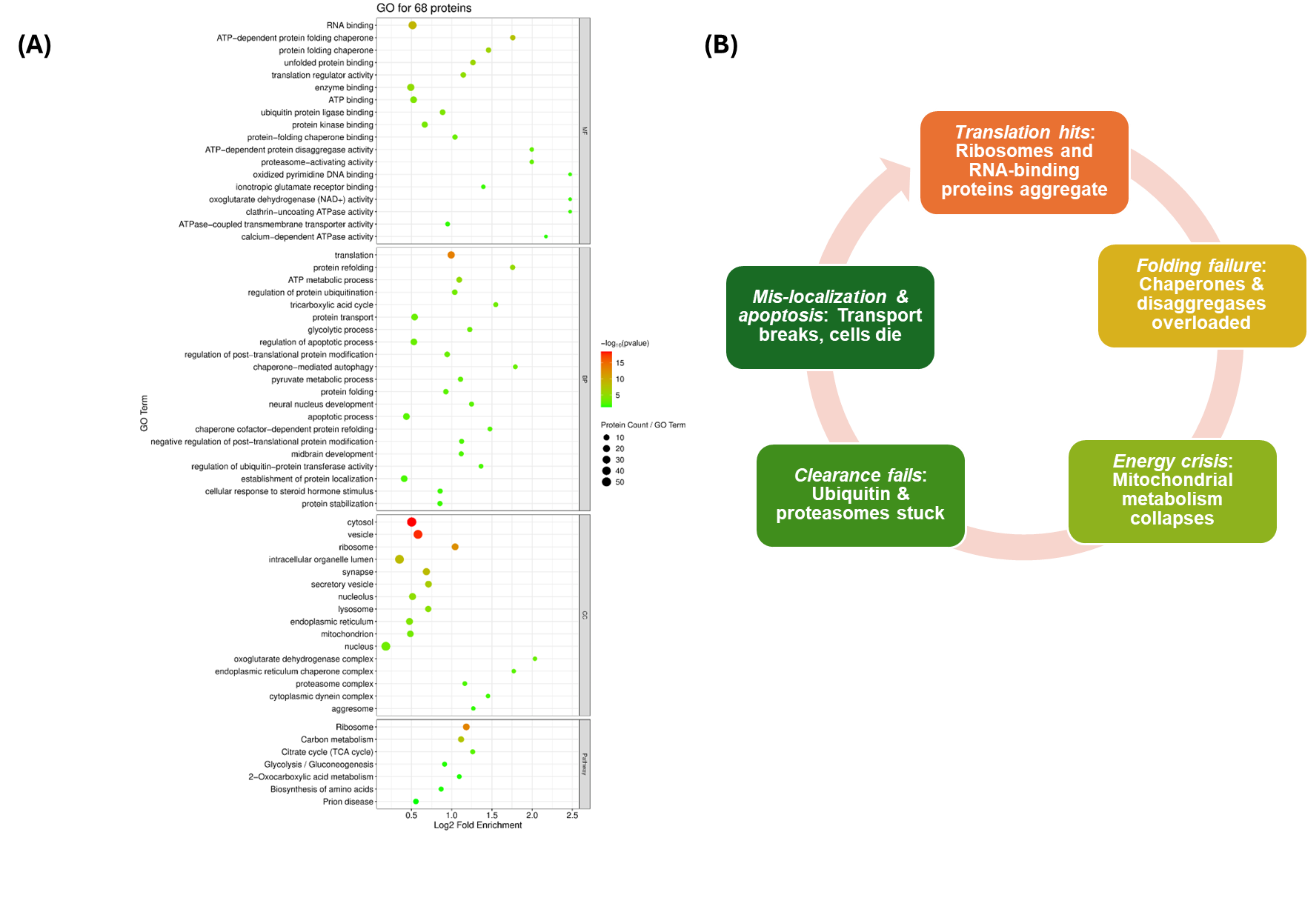

Supplement: Supplementary file 9 [file Image_6.png]

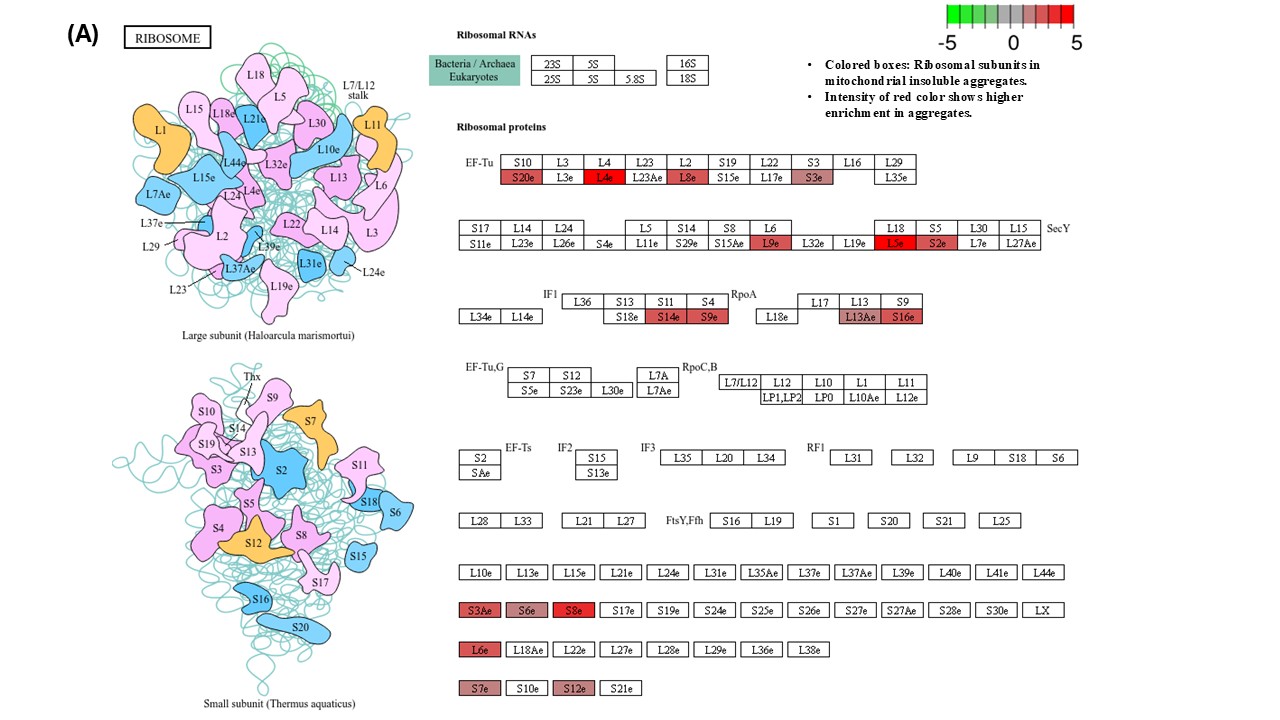

Supplement: Supplementary file 10 [file Image_7.jpeg]
